# Characterization of single neurons reprogrammed by pancreatic cancer

**DOI:** 10.1038/s41586-025-08735-3

**Published:** 2025-02-17

**Authors:** Vera Thiel, Simon Renders, Jasper Panten, Nicolas Dross, Katharina Bauer, Daniel Azorin, Vanessa Henriques, Vanessa Vogel, Corinna Klein, Aino-Maija Leppä, Isabel Barriuso Ortega, Jonas Schwickert, Iordanis Ourailidis, Julian Mochayedi, Jan-Philipp Mallm, Carsten Müller-Tidow, Hannah Monyer, John Neoptolemos, Thilo Hackert, Oliver Stegle, Duncan T. Odom, Rienk Offringa, Albrecht Stenzinger, Frank Winkler, Martin Sprick, Andreas Trumpp

**Affiliations:** 1 Heidelberg Institute for Stem Cell Technology and Experimental Medicine (HI-STEM), Heidelberg, Germany; 2 Division of Stem Cells and Cancer, German Cancer Research Center (DKFZ) and DKFZ-ZMBH Alliance, Heidelberg, Germany; 3 Faculty of Biosciences, Heidelberg University, Heidelberg, Germany; 4 Department of Internal Medicine V, Hematology, Oncology and Rheumatology, Heidelberg University Hospital, Heidelberg, Germany; 5 German Cancer Consortium (DKTK), DKFZ, Heidelberg, Germany; 6 Nikon Imaging Center, University of Heidelberg, Heidelberg, Germany; 7 Single-cell Open Lab, DKFZ, Heidelberg, Germany; 8 German Cancer Research Center (DKFZ), Heidelberg, Germany; 9 Department of Pathology, Heidelberg University Hospital, Heidelberg, Germany; 10 Department of General, Visceral and Transplantation Surgery, Heidelberg University Hospital, Heidelberg, Germany; 11 Department of Neurology and National Center for Tumor Diseases (NCT), Heidelberg University Hospital, Heidelberg, Germany

**Keywords:** Pancreatic cancer, Preclinical research, Pancreas, Pancreatic cancer, Peripheral nervous system

## Abstract

The peripheral nervous system (PNS) orchestrates organ function in health and disease. Most cancers, including pancreatic ductal adenocarcinoma (PDAC), are infiltrated by PNS neurons, and this contributes to the complex tumour microenvironment (TME)^1,2^. However, neuronal cell bodies reside in various PNS ganglia, far from the tumour mass. Thus, cancer-innervating or healthy-organ-innervating neurons are lacking in current tissue-sequencing datasets. To molecularly characterize pancreas- and PDAC-innervating neurons at single-cell resolution, we developed Trace-n-Seq. This method uses retrograde tracing of axons from tissues to their respective ganglia, followed by single-cell isolation and transcriptomic analysis. By characterizing more than 5,000 individual sympathetic and sensory neurons, with about 4,000 innervating PDAC or healthy pancreas, we reveal novel neuronal cell types and molecular networks that are distinct to the pancreas, pancreatitis, PDAC or melanoma metastasis. We integrate single-cell datasets of innervating neurons and the TME to establish a neuron–cancer–microenvironment interactome, delineate cancer-driven neuronal reprogramming and generate a pancreatic-cancer nerve signature. Pharmacological denervation induces a pro-inflammatory TME and increases the effectiveness of immune-checkpoint inhibitors. The taxane nab-paclitaxel causes intratumoral neuropathy, which attenuates PDAC growth and, in combination with sympathetic denervation, results in synergistic tumour regression. Our multi-dimensional data provide insights into the networks and functions of PDAC-innervating neurons, and support the inclusion of denervation in future therapies.

## Abstract (web summary)

A technique that combines retrograde axon tracing with single-cell transcriptomics is used to characterize neurons innervating pancreatic ductal adenocarcinoma and healthy pancreas, providing insight into the role of neural connections in cancer progression.

## Main

The TME mediates tumour development, progression and therapy resistance and provides targets for treatment strategies^3–8^. The role of tumour-infiltrating peripheral neurons in non-neuronal cancers is undisputed, but analyses of their interactions with cancer cells have been restricted to nerve endings^9,10^. The global molecular programs adapted by neurons in contact with cancer cells remain elusive. This is because the perikarya of PNS neurons are located outside the tumour mass, and therefore their molecular information is absent from all currently available bulk or single-cell (sc) RNA-sequencing (RNA-seq) datasets^10^. Several studies have examined the heterogeneous functional relevance of neuronal subtypes for cancer progression in PDAC, and found that dorsal root ganglion (DRG) sensory and celiac ganglion (CG)-derived sympathetic neurons promote, whereas cholinergic vagal activity slows, PDAC growth^11–19^. Hallmarks of PDAC include its desmoplastic stromal ecosystem, which is formed by various cell types and exhibits neural hypertrophy and perineural invasion^1,2^. Neuronal hypertrophy correlates with tumour progression, metastasis and pain, but the underlying mechanisms are unclear^20,21^.

## Various neuronal types innervate the pancreas

**Fig. 1:**
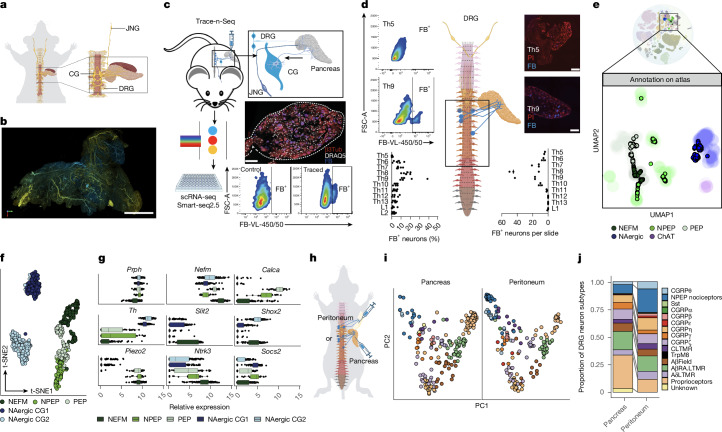
Characterization of pancreas-innervating neurons through Trace-n-Seq. **a**, Schematic of pancreatic neuroanatomy, **b**, Representative LSFM images of an iDISCO-cleared pancreas stained with a pan-neuronal marker (PRPH). Scale bar, 1,000 μm. **c**, Trace-n-Seq workflow. Scale bar, 100 μm. **d**, FACS (*n* = 6) and microscopy (*n* = 3) based quantification of FB^+^ neurons per DRG from thoracic (Th) 5 to lumbar (L) 2 ganglia after intrapancreatic injection of FB. Cell nuclei were visualized with propidium iodide (PI; red). Scale bars, 100 μm. **e**, Projection of Trace-n-Seq of pancreas-innervating neurons onto the PNS atlas of the mouse nervous system^26^. NEFM, peripheral sensory neurofilament neurons; NPEP, peripheral sensory non-peptidergic neurons; PEP, peripheral sensory peptidergic neurons; NAergic, sympathetic noradrenergic neurons; ChAT, cholinergic neurons. **f**,**g**, *t*-SNE plot from Trace-n-Seq of pancreas CG and DRG neurons (**f**) and relative expression of marker genes enriched in subclusters (**g**). Box plots show median and 25% and 75% quantiles, whiskers are 1.5× interquartile ranges, *n* = 633 cells. **h**, Schematic: Trace-n-Seq of peritoneum and pancreas. **i**, *t*-SNE plot of pancreas DRG neurons and peritoneum DRG neurons. **j**, Proportions of neuronal subtypes annotated on the basis of a previously published atlas^27^ within pancreas- or peritoneum-innervating neurons. Bar plots represent data from 151 cells (pancreas) and 181 cells (peritoneum). Source Data

**Extended Data Fig. 1:**
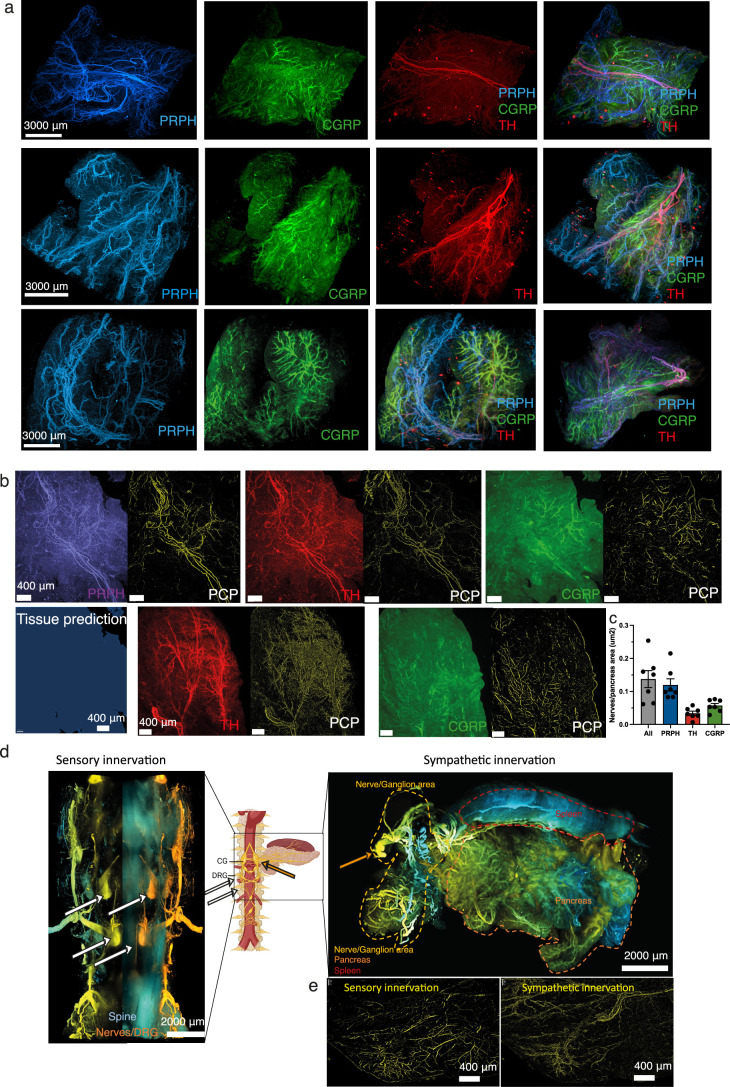
Three-dimensional landscape of pancreatic innervation. **a**, Representative images of nerve axons in whole pancreata obtained using LSFM stained by pan-neuronal marker PRPH (blue), sympathetic marker TH (red), sensory marker CGRP (green). **b**, Quantification of neuronal innervation using machine-learning algorithm. PRPH (violet), TH (red), CGRP (green) positive staining was quantified in a 3D to 2D algorithm and neuronal content was predicted (PCP yellow). Tissue area was predicted based on the autofluorescence of the tissue (dark blue). **c**, PCP based quantification of PRPH+, TH+, CGRP+ nerve /pancreas area (n = 7 mice). **d**, Images of the spine including DRG ganglia containing neuronal cells/axon stained with pan-neuronal marker PRPH obtained using LSFM. Spine is projected (blue) and nerve area (orange and yellow), LSFM image of the CG area, pancreas and spleen. Areas are marked in orange (ganglion area), pancreas (light red) and spleen (dark red). **e**, Representative quantification of neuronal innervation using machine-learning algorithm of sympathetic and sensory axons in the pancreas. Positive staining was quantified in a 3D to 2D algorithm and neuronal content was predicted (PCP yellow). Source Data

**Extended Data Fig. 2:**
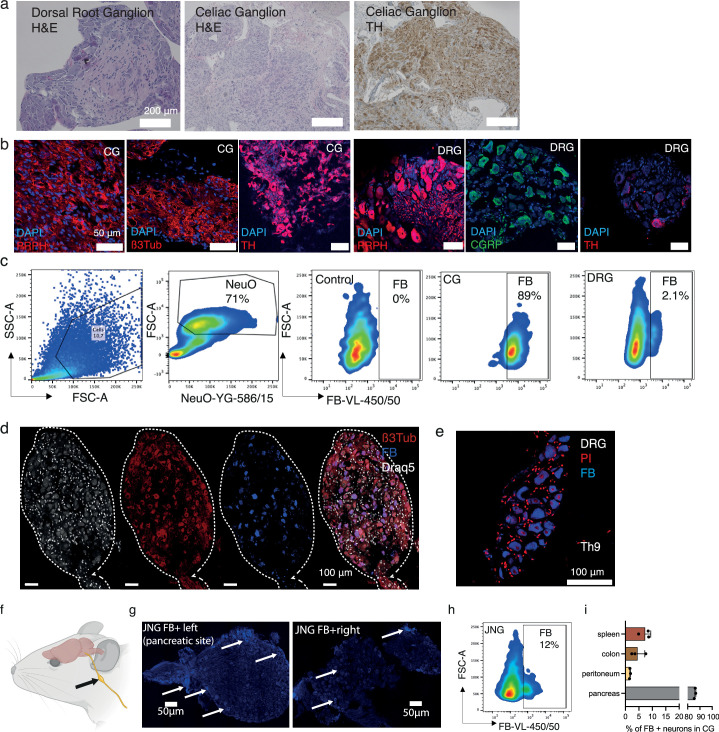
Validation of Trace-n-Seq labelling. **a**, H&E of DRG and CG section and IHC of CG stained with TH. **b**, Representative IF staining of CG stained either for PRPH, TUBB3 or TH, and DRG stained either for PRPH, CGRP or TH. DAPI serves as a nuclear staining. **c**, FACS scheme of retrogradely labelled neurons. Neurons are enriched with the probe NeuO before gating on FB^+^ cells. **d**, Retrogradely labelled cells in a CG 7 days after FB (blue) injection in the pancreas co-stained with DRAQ5 (nuclear dye, white) and TUBB3 (pan-neuro marker, red), scale bar 100 µm. **e**, Retrogradely labelled cells in the dorsal root ganglion 7 days after FB injection in the pancreas co-stained with PI, scale bar 100 µm. **f**, Schematic of pancreatic neuroanatomy of the jugular nodose ganglion (JNG) as part of the vagus nerve. **g**, Retrogradely labelled cells in the jugular nodose ganglion 10 days after FB injection in the pancreas of both sites. Arrows mark positive cells. **h**, FB^+^ tracing efficiency in the JNG after injection in the pancreas analysed by FACS. **i**, FB^+^ tracing efficiency per CG after injection in the pancreas, peritoneum colon and spleen analysed by FACS, n = 3. Source Data

To study the extent and type of neurons present in the mouse pancreas in three dimensions, we imaged iDISCO-cleared samples by light-sheet fluorescence microscopy (LSFM)^22^. Neurons were labelled for peripherin (PRPH, pan-neuron), tyrosine hydroxylase (TH, sympathetic neurons) and calcitonin gene-related peptide (CGRP, sensory neurons), and were quantified using pixel classification prediction (PCP) (Fig. 1a,b, Extended Data Fig. 1 and Supplementary Videos 1–3). In the pancreas, sensory DRG neurons showed a more homogeneous distribution than sympathetic CG neurons, and diverse neuronal subtypes were seen in DRG and CG sections (Extended Data Figs. 1 and 2a,b).

Current scRNA-seq and reference maps of healthy and malignant tissues lack molecular data for innervating neurons, because the cell bodies of these neurons are located outside of their target organs. To address this knowledge gap, we developed a method called Trace-n-Seq (Fig. 1c and Methods). In brief, the fluorescent retrograde tracer Fast Blue (FB) is intraoperatively injected into the target organ^23,24^. FB is taken up by peripheral neurons that innervate the organ, and is retrogradely transported back to the perikarya of the neurons in the PNS ganglia (Fig. 1c). After 5–14 days of tracing, ganglia are removed, digested and stained to isolate single, vital FB^+^ neurons by fluorescence-activated cell sorting (FACS), and subjected to scRNA-seq (Fig. 1c and Extended Data Fig. 2c).

To benchmark the Trace-n-Seq data, we compared FB labelling efficiency in DRGs and CGs by flow cytometry or immunofluorescence microscopy. Both methods revealed that 75–90% of CG neurons and 4–15% of DRG neurons from thoracic ganglia (Th) 6–Th10 projected into the pancreas (Fig. 1d). For the jugular nodose ganglion (JNG), which contains viscero-sensory vagal fibres, around 12% were labelled by FB, similarly to previously reported cholera toxin β tracing data^25^. FB injections into the spleen, colon and peritoneum revealed organ-specific frequencies of labelled neurons, confirming the specificity of Trace-n-Seq (Extended Data Fig. 2c–i).

**Extended Data Fig. 3:**
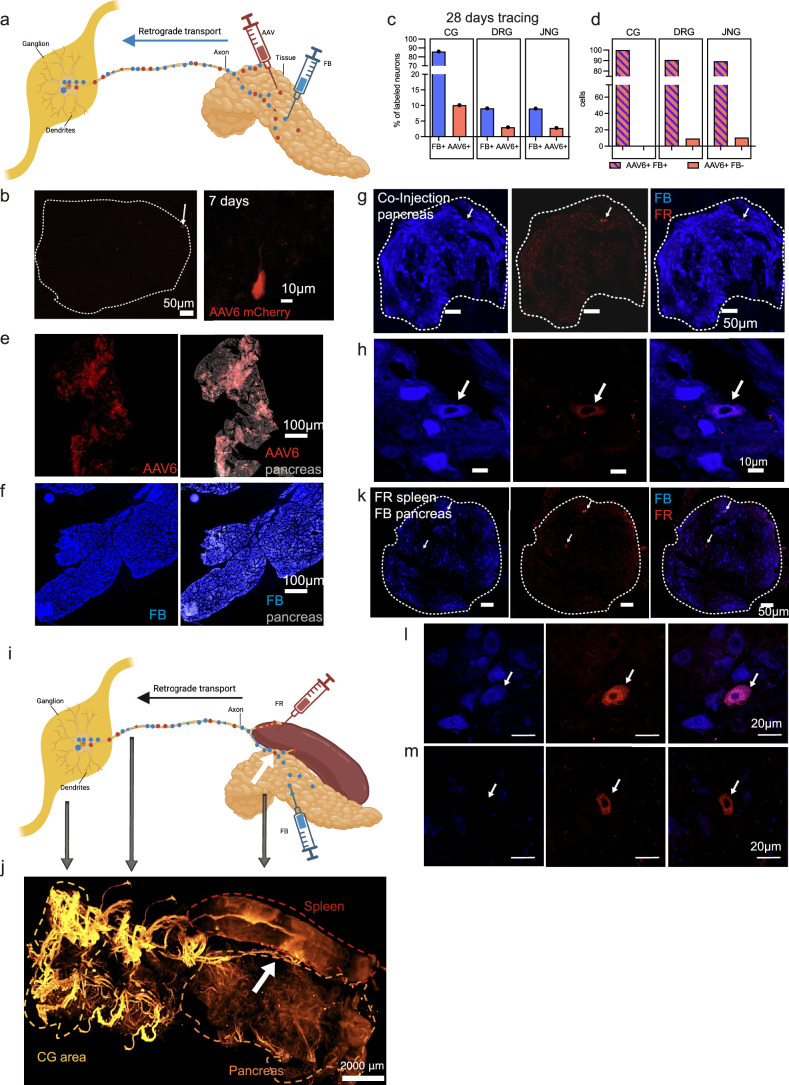
Comparison of retrograde tracer efficiency. **a**, Schematic of intrapancreatic labelling using FB and AAV6. **b**, retrogradely labelled cells in a CG 7 days after AAV6 (mCherry, red) injection in the pancreas, scale bar 50 µm, and zoom-in on mCherry-positive cell, scale bar 10 µm. **c**, FACS based analysis of AAV6 and FB positive cells per CG after 28 days of tracing. Shown are % of labelled cells per dye; n = 1. **d**, FACS based analysis of AAV6 double (also FB) and single positive cells per total number of AAV positive cells after 28 days of co-tracing. **e**, Image of full pancreas section 10 days after AAV6 injection shows distribution of the tracer throughout the pancreas, mostly positivity is observed at the injection sites. **f**, Image of full pancreas section 10 days after FB injection shows distribution of the tracer throughout the pancreas, universal positivity is observed in the entire organ. **g**, Retrogradely labelled cells in a CGs 7 days after co-injection of FB (blue) and FluoroRuby (FR, red) in the pancreas. **h**, and zoom-in on double-positive (FB+/FR+) cell, marked by white arrow. **i**, Schematic of intrapancreatic and intrasplenic labelling using FB (pancreas) and FR (spleen) with shared nerve axons. **j**, LSMF image of CG-pancreas-spleen area stained with peripherin. Different areas marked as indicated, white arrow points to nerve axon sprouting through pancreas and spleen. **k**, Retrogradely labelled cells in a CG 7 days after injection of FB (blue) in the pancreas and FluoroRuby (FR, red) into the spleen, scale bar 50 µm. **l**, and zoom-in on double-positive (FB+/FR+) cell, sprouting through pancreas and spleen, marked by white arrow, scale bar 20 µm. **m**, and zoom-in on single-positive (FR+) cell, traced from spleen, not projecting through pancreas, marked by white arrow, scale bar 20 µm.

Alternative tracers, including adeno-associated viruses (AAVs) and FluoroRuby (FR), showed lower efficiencies than FB. Intrapancreatic co-injection of FB with AAV6 revealed co-labelling of 95% of AAV6^+^ neurons with FB. Similarly, co-injection of FB with FR allowed dual labelling of neurons, but FB had a superior labelling efficiency; we therefore used FB in this study (Extended Data Fig. 3a–h). By simultaneous pancreatic FB and splenic FR injections, we discovered that single CG neurons innervate both organs, and this finding was supported by three-dimensional (3D) imaging (Extended Data Fig. 3i–m and Supplementary Video 1).

## Dissecting pancreas neuronal heterogeneity

**Extended Data Fig. 4:**
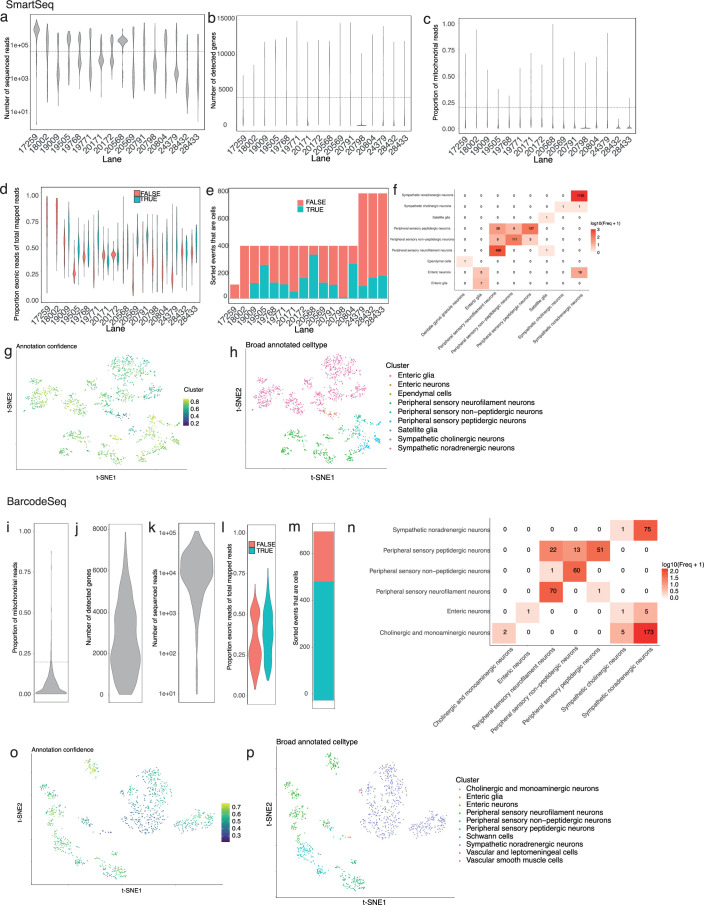
Quality control of scRNA-seq data generated by Trace-n-Seq. Smart-seq quality was assessed among different sequencing lanes by. **a**, Number of obtained reads/ lane. **b**, Number of detected genes/cell. **c**, Proportion of mitochondrial reads. **d**, Proportion of exonic reads of total mapped genes, **e**, Identifying events that are cells. **f**, Annotation efficiency. **g**, Annotation confidence according to a previous report^26^. **h**, Annotation of broad cell types. Barcode-seq quality was assessed among sequencing lanes by. **i**, Proportion of mitochondrial reads. **j**, Number of detected genes/cell. **k**, Number of sequenced reads. **l**, Proportion of exonic reads of total mapped genes, **m**, Identifying events that are cells. **n**, Annotation efficiency. **o**, Annotation confidence according to a previous report^26^. **p**, Annotation of broad cell types.

**Extended Data Fig. 5:**
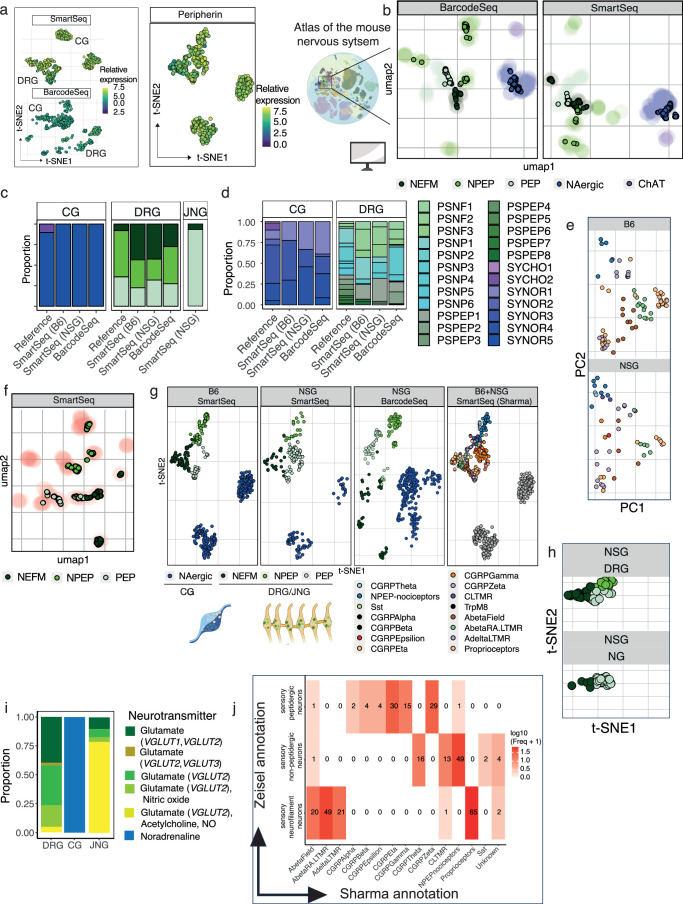
Atlas-based cell-type annotation of Trace-n-Seq data. **a**, t-SNE plots comparing relative expression of *Prph* in Smart-seq and Barcode-seq-based scRNA-seq of pancreas-innervating CG and DRG and t-SNE plots of Smart-seq-based scRNA-seq of pancreas-innervating CG, JNG and DRG neurons. **b**, Projection of our dataset of pancreas-innervating neurons onto the PNS atlas of the mouse nervous system^26^ separated for the Smart-seq and Barcode-seq dataset. **c**, Neuronal subtype abundance of pancreatic sympathetic (CG) and sensory (DRG, JNG) neurons compared to a reference atlas^26^. **d**, In-depth neuronal subtype proportion of sympathetic and sensory neurons in the pancreas (BL6, NSG, Smart-seq and Barcode-seq) compared to the full PNS reference^26^. **e**, PC plot from Trace-n-Seq of NSG and BL6 DRG neurons with clusters annotated based on a previous report^27^. **f**, Projection of our dataset of pancreas-innervating DRG neurons onto a previously published DRG atlas^27^. **g**, t-SNE plot from Trace-n-Seq of pancreas CG and DRG neurons with clusters annotated based on previous reports^26,27^. **h**, t-SNE of DRG and JNG sensory neurons in the pancreas annotated in a previous report^26^. **i**, Neurotransmitter status of pancreas CG, JNG and DRG neurons, **j**, Overlay of DRG subtypes between broad annotation in previous studies.

After establishing Trace-n-Seq, we systematically analysed healthy pancreas-innervating CG, DRG and JNG neurons from NSG and C57/BL6 (BL6) mice. In total, we analysed 1,384 high-quality single neurons for downstream analysis (589 DRG, 689 CG and 106 JNG; Extended Data Fig. 4). All cells were bona fide neurons expressing *Prph*, and neuronal subtypes were annotated with a scRNA-seq atlas of the mouse nervous system^26^ (Fig. 1e and Extended Data Fig. 5a,b). This analysis revealed that the pancreas was innervated by two major sympathetic, exclusively noradrenergic (NAergic) neuronal subtypes (CG1 and CG2) and three previously described JNG- or DRG-derived glutamatergic neuronal subtypes: (1) neurofilament (NEFM), (2) peptidergic (PEP) and (3) non-peptidergic (NPEP) neurons^26^ (Fig. 1f,g and Extended Data Fig. 5b–d).

DRG cell types can be subclassified into 17 DRG subtypes, of which only 12 projected into the pancreas. All five undetected subtypes were classic nociceptive neurons (Extended Data Fig. 5d). To increase robustness, DRG neurons were annotated using a second atlas^27^. This enabled us to detect functionally equivalent populations with similar frequencies of the three main subtypes of DRG neurons in NSG and BL6 mice. By contrast, JNG neurons were mostly classified as NPEP (ref. ^28^) (Extended Data Fig. 5e–j).

**Extended Data Fig. 6:**
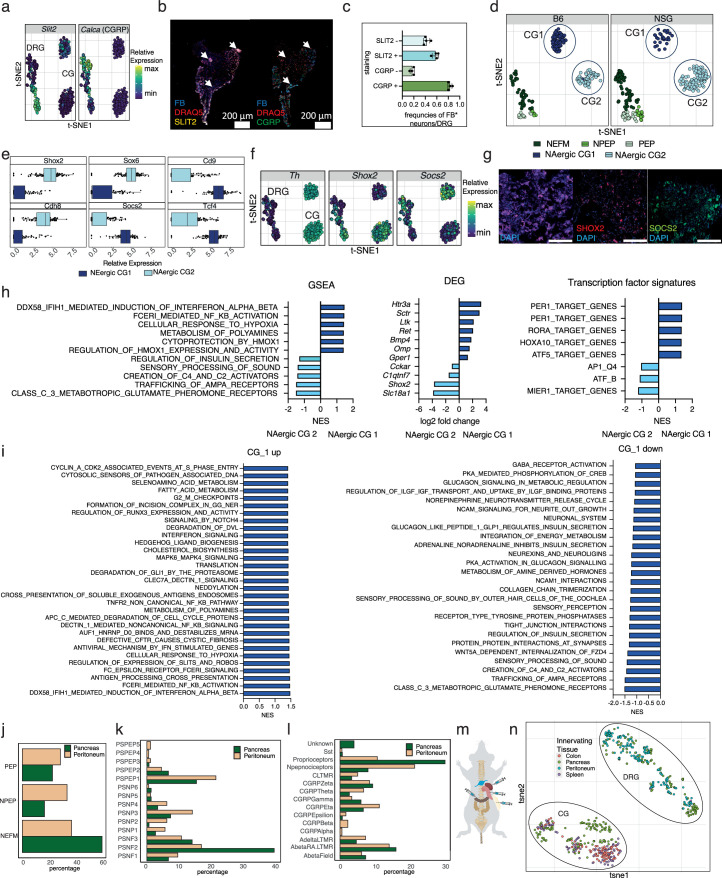
Identification of neuronal subtypes in peripheral ganglia. **a**, Relative expression of *Slit2* and *Calca* defining DRG clusters. **b**, IF staining of DRG ganglia for SLIT2 (yellow) and CGRP (green) scale bar 200 µm. **c**, Quantification of FB^+^, SLIT2 or CGRP labelled neurons in DRG n = 3 mice. **d**, t-SNE plot from Trace-n-Seq of pancreas CG and DRG neurons (BL6/ NSG). **e**, Relative expression marker genes enriched in CG subclusters. The box plots show median and 25%- and 75%-quantiles, the whiskers 1.5 interquartile ranges. **f**, Relative expression of *Th*, *Shox2* and *Socs2* defining CG subclusters. **g**, IF staining of CG section with TH, SHOX2, SOCS2; scale bar 100 µm. **h**, GSEA, differential expression and transcription factor analysis between clusters NAergic CG1 and NAergic CG2. **i**, GSEA between CG Cluster 1 and Cluster 2 (shown as CG1 up/down, down in CG1 means up in CG2). **j**, Broad subtype proportion of sensory pancreas neurons compared to sensory peritoneum neurons annotated in a previous study^26^, **k**, In-depth subtype proportion of sensory pancreas neurons compared to sensory peritoneum neurons annotated in a previous study^26^. **l**, Subtype proportion of sensory pancreas neurons compared to sensory peritoneum neurons annotated in a previous report^27^. **m**, Schematic of retrograde labelling of spleen- pancreas- colon- and peritoneum neurons. **n**, t-SNE of scRNA-seq (Smart-seq, NSG) of 102 pancreas, 181 peritoneum, 76 spleen and 128 colon innervating neurons. Source Data

At the individual gene level, DRG but not CG neurons expressed *Piezo2* and DRG subtypes were characterized by *Slit2* (NEFM), *Calca*, *Bmpr1b* (PEP) or *Socs2* (NPEP) expression. Selective SLIT2 and CGRP (*Calca*) expression in pancreas FB-traced DRG neurons was validated by immunofluorescence microscopy (Fig. 1g and Extended Data Fig. 6a–c).

Owing to the lack of a CG atlas, CG neurons were annotated on the basis of sympathetic trunk data^26^. Three out of five NAergic but no sympathetic cholinergic neurons were detected in the CG (ref. ^23^) (Extended Data Fig. 5c,d). The two CG main subclusters exclusively expressed *Shox2* (cluster 1) or *Socs2* and *Slit2* (cluster 2) at the RNA and the protein level (Fig. 1g and Extended Data Fig. 6d–g). Similar to sympathetic trunk, cluster 1 contained neurons that regulate piloerection and vasoconstriction. Cluster 2 did not match described subtypes but expressed markers of cholinergic sympathetic neurons that regulate sweat glands. Gene set enrichment analysis (GSEA) showed increased glutamatergic receptor expression and ‘regulation of insulin secretion’, suggesting a role in endocrine control. In addition, targets of the transcription factors AP-1 and MIER1 were enriched^23^ (Extended Data Fig. 6h,i). In cluster 1, the upregulation of gene sets associated with hypoxia, HMOX1 signalling and transcription was driven by *Per1*, *Hoxa10* and *Atf5*, which are known to modulate circadian rhythm^29^ (Extended Data Fig. 6h,i).

To study organ specificity, we applied Trace-n-Seq to the peritoneum (181 DRG neurons), transverse colon (128 CG neurons) and spleen (76 CG neurons). This revealed unique neuronal subtype frequencies: more CGRPα^+^, CGRPβ^+^ and CGRPε^+^ PEP nociceptors innervated the peritoneum, and more NEFM neurons innervated the pancreas (Fig. 1h–j and Extended Data Fig. 6j–l). Although the composition of spleen-, colon- and pancreas-innervating CG neurons was similar, we observed differentially expressed genes (DEGs) in neurons from the same ganglia projecting into different organs (Extended Data Fig. 6m,n). In summary, Trace-n-Seq enables scRNA-seq of hundreds of neurons innervating any tissue. Our data confirm and expand textbook neuroanatomy by transcriptional profiling. This includes increased nociceptive innervation in the peritoneum compared with epithelial abdominal organs, and the absence of sweat-gland-innervating cholinergic sympathetic neurons in the CG. Trace-n-Seq can provide notable molecular insights into organ innervation to study diverse physiological or pathological conditions.

## Neuronal hyperinnervation in PDAC

**Fig. 2:**
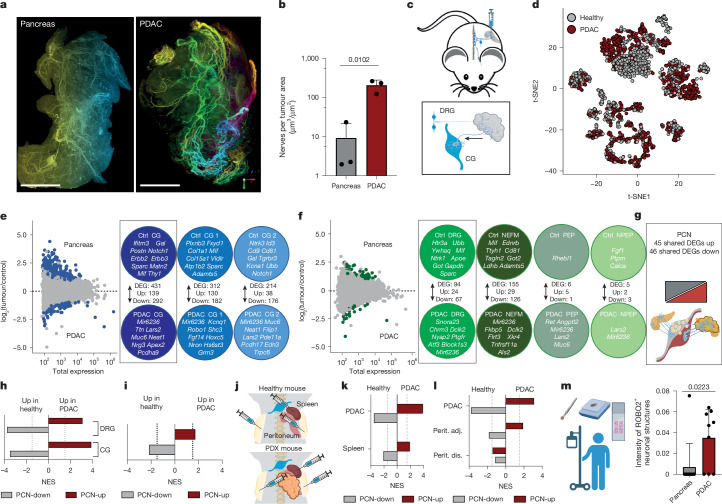
PDAC induces a tumour-specific neuronal expression profile. **a**, Representative images of PRPH-stained pancreas and tumour innervation obtained using LSFM, Scale bars, 1,000 μm. **b**, Neuronal sprouting quantified in PCP-transformed LSFM images: total nerve area/tissue area in full pancreas (*n* = 3 mice) and full PDAC (*n* = 3 mice). Two-tailed unpaired *t*-test. Mean ± s.d. **c**, Schematic: Trace-n-Seq in PDAC. **d**, *t*-SNE plot of pancreas versus PDAC neurons. **e**,**f**, Volcano plots showing number of and exemplary DEGs between pancreas and PDAC in all CG neurons and subclusters (**e**) and in all DRG neurons and subclusters (**f**). Genes are considered to be significantly differentially expressed at an adjusted *P* value of less than 0.1 (DESeq Wald test, Benjamini–Hochberg correction for multiple testing). Ctrl, control. **g**, Schematic description of PCN-up and PCN-down signatures. **h**,**i**, Enrichment analysis of PCN-up and PCN-down signatures for DEGs between pancreas and PDAC in CG and DRG neurons (**h**) and for DEGs between pancreas and PDAC (PDX) JNG neurons (**i**). NES, normalized enrichment score. **j**, Experimental outline: Trace-n-Seq of neurons traced from healthy mice (spleen and peritoneum) or PDX mice (spleen and peritoneum: tumour-adjacent and tumour-distant). **k**,**l**, Enrichment analysis of PCN-up and PCN-down signatures comparing CG neurons traced from pancreas versus PDAC, or spleens of healthy versus PDAC (PDX) mice (**k**), and DRF neurons from pancreas versus PDAC, or peritoneum (healthy versus tumour-adjacent or tumour-distant) (**l**). **m**, ROBO2 intensity (gene from PCN-up signature) in nerves innervating the healthy human pancreas or PDAC (*n* = 11 and *n* = 9 patients). Two-tailed unpaired *t*-test. Mean + s.d. Source Data

**Extended Data Fig. 7:**
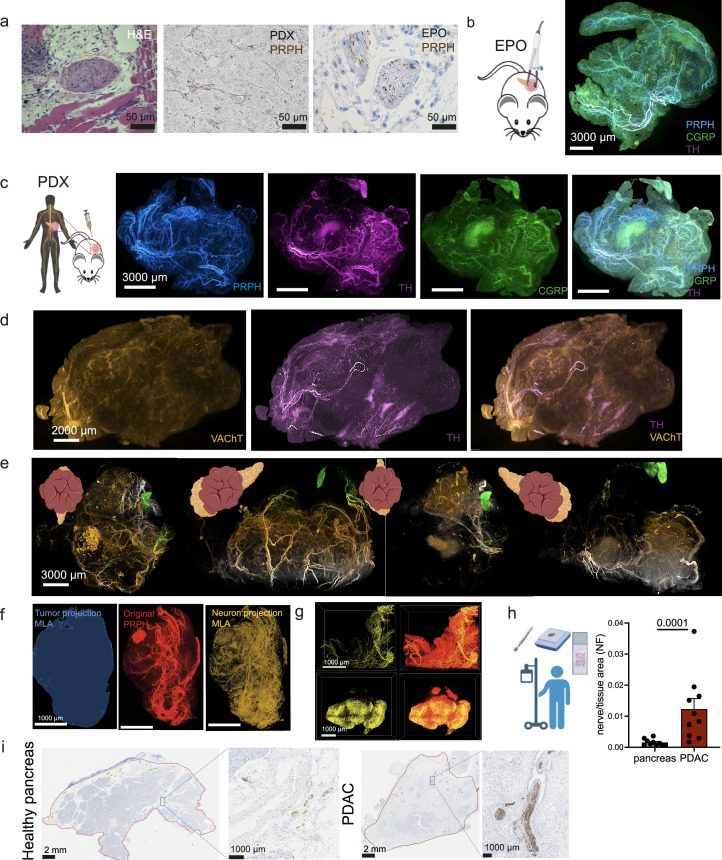
Imaging of neuronal infiltration in PDAC. **a**, H&E of PDAC (PDX- PACO10) specimen, IHC of PDX and EPO specimen stained for pan-neuronal marker PRPH. **b**, EPO model schematic and image by LSFM. **c**, Schematic of intrapancreatic PDX model and representative images of tumour innervation obtained using LSFM. PRPH (blue), TH (violet), CGRP (green). **d**, Representative images of tumour innervation obtained using LSFM. TH (violet), VAChT (yellow). **e**, Representative images of tumour innervation obtained using LSFM from four different angles of the tumour. **f**, Representative images of quantification of neuronal innervation using PCP. Tumour area was predicted using autofluorescence. Positive staining (red) was quantified in a 3D algorithm and neuronal content was predicted (Yellow). **g**, PCP prediction of nerves (yellow) in pancreas specimen imaged by LSFM with (red) and without tissue prediction (upper part) and PCP prediction of nerves (yellow) in PDAC specimen imaged by LSFM with (red) and without tissue prediction (lower part). **h**, Quantification of nerve/tissue area of matched human pancreas (grey) and treatment naive PDAC in (red) NF stained IHC slides (n = 10). Mann–Whitney test. Mean +/− SEM. **i**, Images of stained tissues of NF1 stained IHC slides. Source Data

**Extended Data Fig. 8:**
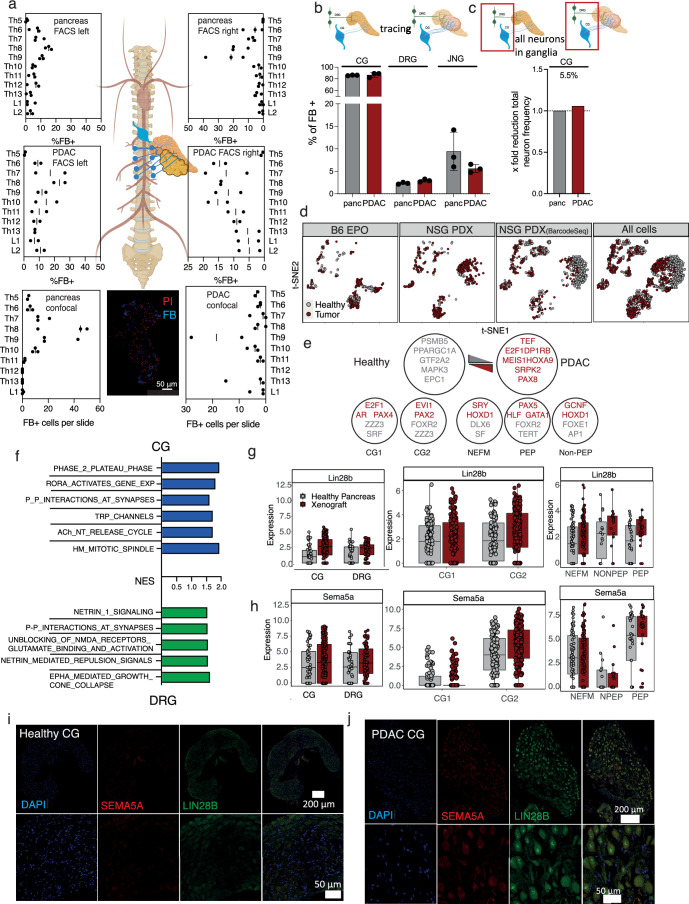
Neuronal reprogramming in PDAC neurons. **a**, FACS and microscopy based quantification of FB^+^ DRG neurons. Pancreas/PDAC innervation after retrograde labelling of DRG (single) analysed by FACS (left and right) and IF confocal microscopy (n = 3). **b**, FACS based quantification of FB^+^ DRG, JNG and CG neurons. Pancreas/PDAC innervation after retrograde labelling of DRG (Th5-L1 combined) analysed by FACS microscopy (n = 3). Two-tailed unpaired t-test PDAC vs healthy traced neurons: ns for all comparisons. **c**, Proportion of total number of neurons (mean of NeuO+ cells per CG) pancreas/PDAC (n = 3). **d**, t-SNE plot of pancreas vs. PDAC neurons in by Smart-seq (BL6 and PDX) and Barcode-seq. **e**, GSEA analysis of transcription factor signatures deregulated in pancreas/PDAC subclusters. **f**, GSEA analysis of top upregulated gene sets in PDAC in detected CG or DRG. **g**, Gene expression of selected DEGs: *Lin28b* between pancreas and PDX CG and DRG neurons in total or for the separate neuronal subtypes. All box plots in this figure show median, 25%- and 75%-quantiles, whiskers 1.5 interquartile ranges and represent data from 333 (pancreas) and 605 (tumour) cells. **h**, Gene expression of selected DEGs *Sema5a* between pancreas and PDAC CG and DRG neurons in total or for the separate neuronal subtypes. **i**, Validation of sequencing results by IF staining of healthy CG of LIN28B and SEMA5a. **j**, Validation of sequencing results by IF staining of PDAC CG of LIN28B and SEMA5a. Source Data

Next, we studied neurons infiltrating four different PDAC models: two human orthotopic PDAC xenograft (PDX) models^30,31^; an autochthonous model based on in vivo electroporation of a *KRAS-G12V* transposon and CRISPR–Cas9-mediated *Tp53* deletion (EPO model, Methods); and an allograft model using a KPC-mouse-derived cell line. Because quantification of neuronal networks using standard two-dimensional (2D) histology is limited, we assessed PDAC innervation by 3D LSFM of cleared tumours (Extended Data Fig. 7a). Nociceptive (CGRP), sympathetic (TH) and parasympathetic (vACHT) neurons exhibited disorganized sprouting with axon terminals densely spread throughout the entire tumour mass, omitting necrotic regions (Fig. 2a, Extended Data Fig. 7b–g and Supplementary Video 4). In PDAC, neurons covered a greater area than they did in healthy pancreas, as observed in human PDAC (Fig. 2b and Extended Data Fig. 7h,i). To study this increase, we injected FB into PDX tumours. Total FB^+^ neurons per ganglion and total neurons per ganglion were comparable to the numbers observed for healthy pancreas, suggesting increased axonal sprouting of pre-existing neurons. We detected more DRG-L1 neurons in PDAC, probably owing to attraction from nearby organs during tumour growth (Extended Data Fig. 8a–c). Therefore within our models, hyperinnervation in PDAC arose from axonal sprouting rather than from neurogenesis.

## PDAC reprograms innervating neurons

To identify PDAC-induced changes, we integrated our Trace-n-Seq data of PDAC and pancreas neurons. We identified clusters similar to the healthy setting in all PDAC models (Fig. 2c,d and Extended Data Fig. 8d). By comparing 354 healthy and 960 PDAC CG neurons, we identified 431 DEGs (139 up and 292 down; cluster 1: 312, cluster 2: 214; Fig. 2e). CG PDAC neurons downregulated genes encoding neuropeptides (*Gal*), receptors (*Erbb2*, *Erbb3* and *Ntrk3*), extracellular matrix proteins (*Matn2*, *Postn* and *Sparc*) or the immune regulator *Mif*. Vice versa, PDAC induced the expression of the axon guidance receptor *Robo1*, non-coding RNAs (*Mir6236*, *Neat1* or *Lars2*), transcription factors (*Hoxc5*) or signalling molecules (*Fgf14* and *Edn3*) (Fig. 2e). GSEA for all CG data revealed enrichment for processes such as calcium signalling, synapse formation and microtubule assembly in PDAC neurons, consistent with increased axonal sprouting (Extended Data Fig. 8e,f).

**Extended Data Fig. 9:**
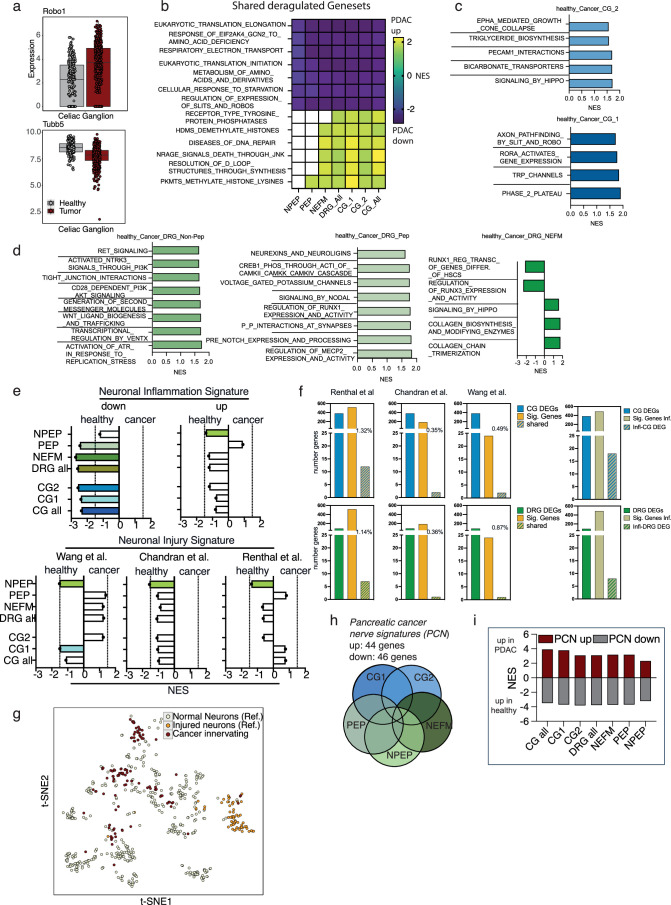
Functional signature in PDAC neurons. **a**, Relative gene expression of DEG *Robo1* and *Tubb5* between pancreas and PDAC CG neurons. **b**, GSEA analysis: Most commonly shared from top 20 up- and downregulated gene sets between subclusters. **c**, GSEA between pancreas and PDAC CG neurons specified to CG Cluster 1 and Cluster 2. **d**, GSEA between pancreas and PDAC DRG neurons specified to different neuronal subtypes (NEFM, PEP, NPEP). **e**, GSEA analysis of signatures of neuronal inflammation and injury on DEG analysis of pancreas/PDAC subclusters. Coloured bar had a FDR < 0.05. **f**, Display of number of genes that are DEG in the pancreas-PDAC neuron dataset, the number of genes reported in the published injury or inflammation signatures and the overlay of common genes found in both, for sympathetic CG and sensory DRG neurons. **g**, t-SNE of the injury dataset^39^ overlayed with our analysed PDAC-innervating neurons. **h**, Schematic of shared DEGs between subclusters used for PCN signature. **i**, Enrichment analysis of PCN-up/down signatures of all DRG/ CG neuron subtypes based on Smart-seq.

PDAC DRG neurons upregulated signatures of neuronal guidance factors (*Ntn1* or Epha) and glutamate receptor signalling. Transcription factor analysis identified downregulation of metabolic regulators such as *Ppargc1a*, whereas neuronal development programs such as Hox and *Meis1* were upregulated in PDAC neurons^32–34^ (Extended Data Fig. 8e,f). We identified 94 DEGs with different effects on DRG subtypes. We found 155 DEGs in NEFM, but only 6 DEGs in PEP and 5 DEGs in NPEP neurons (Fig. 2f). The DEGs that showed the lowest expression in PDAC compared with pancreas neurons were related to metabolism (*Gapdh* and *Got1*), neuropeptides (*Calca*), receptors (*Ntrk1* and *Htr3a*) and immune regulators (*Mif*). Genes with higher expression in PDAC neurons included the transcription factors *Atf3* and *Dclk2*, secreted factors (*Angptl2*), receptors (*Ptgfr*) and again the non-coding RNAs *Lars2* and *Mir6236* (Fig 2e). Some factors such as *Lin28b*, a master regulator of stemness, were upregulated at the RNA and protein level in all CG or DRG neurons in the PDX models^35^. *Sema5a* increased only in CG cluster 2 and PEP neurons, highlighting the importance of single-cell resolution (Extended Data Figs. 8g–j and 9a).

To gain deeper insight into PDAC-induced transcriptional changes, we performed GSEA on all subpopulations and the depleted signatures aligned in all tumour settings. All subgroups showed a metabolic switch, with ‘electron transport chain’ or ‘metabolism of amino acids and derivates’ depleted in PDAC neurons. The few shared enriched processes included ‘histone modifications’, ‘resolution of D-loop structures’ and ‘DNA repair’, which correlate with neuronal damage and regeneration in response to malignancy^36^ (Extended Data Fig. 9b–d). Enriched gene sets were most similar between NEFM, CG1 and CG2 neurons, whereas PEP and NPEP neurons were hardly affected (Extended Data Fig. 9b). In summary, Trace-n-Seq revealed changes associated with neuronal development and axon guidance, metabolism and microenvironmental processes such as stromal and immune regulation between PDAC and healthy pancreas neurons.

## PDAC induces a cancer nerve signature

**Extended Data Fig. 10:**
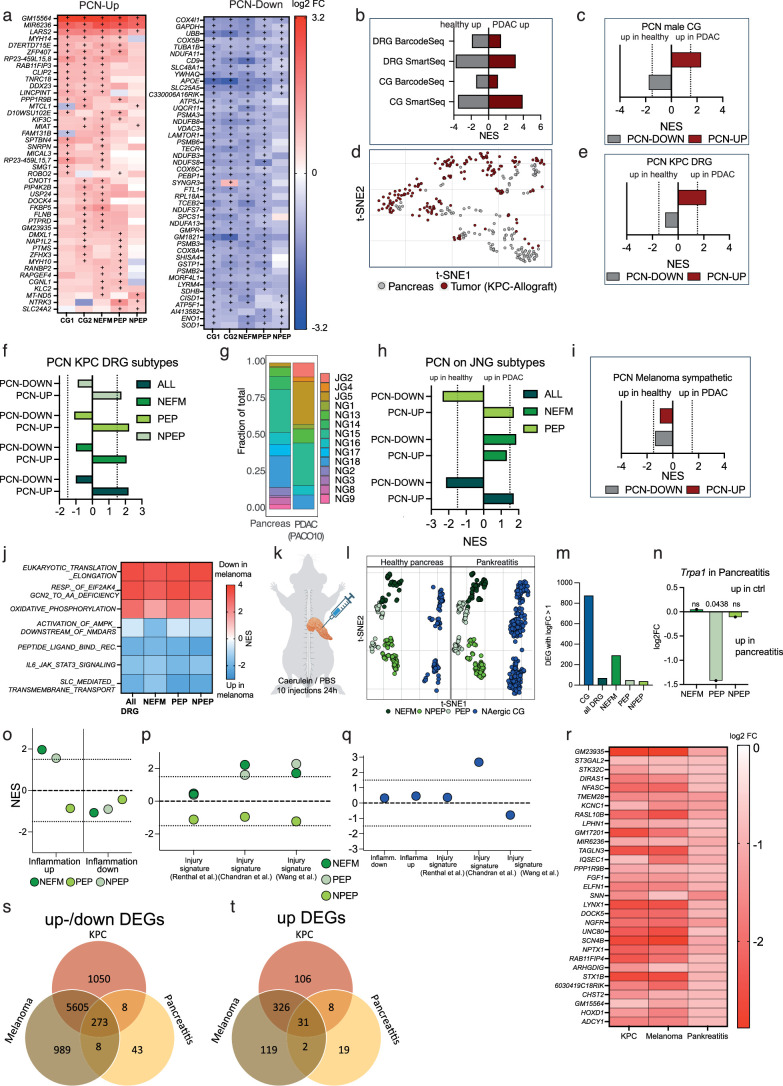
Neuronal expression changes in cancer and pancreatitis. **a**, List of all genes of the PDAC up and down signatures, + indicates whether the gene was detected as differential in the specific neuronal subtype. **b**, Enrichment analysis of PCN-up/down signatures of DRG/ CG neurons based on Barcode-seq. **c**, Enrichment analysis of PCN-up/down signatures of CG neurons in male PDX model. **d**, t-SNE of pancreas and KPC-allograft innervating neurons. **e**, Enrichment analysis of PCN-up/down signatures of DRG neurons in KPC-Allograft model. **f**, Enrichment analysis of PCN-up/down signatures of DRG all, NEFM, PEP, NPEP neurons in KPC-Allograft model. **g**, Neuronal subtypes within the JNG in healthy pancreas and PDAC (PDX) innervating neurons annotated based on ref. ^57^. **h**, Enrichment analysis of PCN-up/down signatures of DRG all, NEFM, PEP neurons in JNG between healthy pancreas NSG vs PDAC (PDX) model. **i**, Enrichment analysis of PCN-up/down signatures of sympathetic neurons in the melanoma model. **j**, GSEA analysis: Most commonly shared up- and downregulated gene sets between subclusters in the melanoma model. **k**, Schematic of acute pancreatitis model in BL6 mice. **l**, t-SNE of healthy pancreas control- and pancreatitis-innervating neurons, cell types annotated in a previous study^26^. **m**, Number of DEGs between healthy pancreas control- and pancreatitis-innervating neurons among the different neuronal subtypes. **n**, *Trpa1* expression in DRG subtypes between healthy control neurons and neurons innervating acute pancreatitis. logFold Change based on control cells and pancreatitis sc- neurons, DESEQ2. **o**, Enrichment analysis of neuronal inflammation signatures on pancreas vs. pancreatitis-innervating DRG neuron subclusters. **p**, GSEA analysis of signatures of neuronal injury on DEG analysis of pancreas/pancreatitis DRG subclusters. **q**, GSEA analysis of signatures of neuronal inflammation and injury on DEG analysis of pancreas/pancreatitis CG neurons. **r**, Heat map of up- or downregulated genes across the different models of KPC, melanoma and pancreatitis compared to their respective healthy controls. **s**, Venn diagram of shared up/downregulated genes in KPC-, melanoma- and pancreatitis model. **t**, Venn diagram of shared upregulated genes in KPC-, melanoma- and pancreatitis model.

We next compared these changes with signatures of neuronal stress from denervation or sterile inflammation^37–40^. PDAC neurons and ‘sterile inflammation’ showed similarities in their downregulated genes, suggesting similar metabolic rewiring (Extended Data Fig. 9e). However, sterile-inflammation-induced genes correlated with PDAC NPEP but not NEFM, PEP or sympathetic neurons. Only minimal similarities were found between injury or inflammation signatures and PDAC DEGs (Extended Data Fig. 9f). By clustering our PDAC neuron with scRNA-seq data from neurons after surgical denervation, we reproduced the formation of a new ‘denervation stress’ cell state. This cluster contained very few PDAC neurons, supporting distinct changes between injury and cancer (Extended Data Fig. 9g). To identify subtype-independent changes in PDAC neurons, we integrated robustly expressed repeatedly differentially expressed genes from all five major CG and DRG subclusters to create two pancreatic-cancer nerve (PCN) signatures (PCN-up, 45 genes; PCN-down, 46 genes) (Fig. 2g, Extended Data Figs. 9h,i and 10a and Supplementary Table 2). We tested PCN-signature enrichment on all CG or DRG neurons together or on their subpopulations. PCN-up was enriched in PDAC neurons, whereas PCN-down was enriched in healthy neurons in every subpopulation, with NPEP scoring the lowest (Fig. 2h and Extended Data Fig. 9i). For validation, signatures were evaluated in four additional models. PCN signatures correctly identified healthy versus PDAC CG and DRG neurons in barcode sequencing (Barcode-seq) data (770 neurons; model 1), and PDAC PDX tumour neurons in male mice (214 neurons; model 2) (Extended Data Fig. 10b,c). PCN-up was also enriched in DRG neurons traced in KPC-allograft PDAC, whereas PCN-down only trended towards control neurons, potentially owing to fast tumour growth (336 neurons; model 3) (Extended Data Fig. 10d–f). PCN signatures also correctly enriched for PDAC and control neurons in JNG neurons (171 neurons; model 4), even though they were developed by analysing CG and DRG neurons (Fig. 2i and Extended Data Fig. 10g,h).

Collectively, we have condensed common molecular traits into a PDAC nerve signature, most prominently seen in NEFM and sympathetic neurons. This allows the detection of PDAC-induced changes in neuronal expression that are distinct to inflammation or neuronal injury.

## PCN signatures depend on tumour proximity

To assess whether PDAC systemically induces the observed signature, we injected FB into orthotopically transplanted PDAC PDX or healthy mice and compared traced neurons from the spleen and from the peritoneum. For PDAC PDX mice, FB was injected at tumour-adjacent (spleen or peritoneum), and tumour-distant sites (pelvic peritoneum) (Fig. 2j). We assessed the PCN signatures and found that PCN-down genes were enriched in healthy and PCN-up genes in PDX spleen neurons (Fig. 2k). Spleen and pancreas are anatomically adjacent, with some neurons innervating both, which suggests that tumours might influence splenic haematopoietic and immune cells through reprogrammed neurons. Similarly, the PCN-up signature was enriched in peritoneum-innervating DRG neurons of PDX mice close to the primary tumour, but not in pelvic DRG neurons (Fig. 2j–l). Notably, this was regionally limited, because no global deregulation of tumour-distant DRG neurons was detected (Fig. 2l). In addition, we investigated the expression of ROBO2 as member of the PCN-up signature in human pancreas and PDAC and validated the upregulation of ROBO2 in human PDAC neurons (Fig. 2m).

## Intrapancreatic cancers attract NEFM neurons

**Fig. 3:**
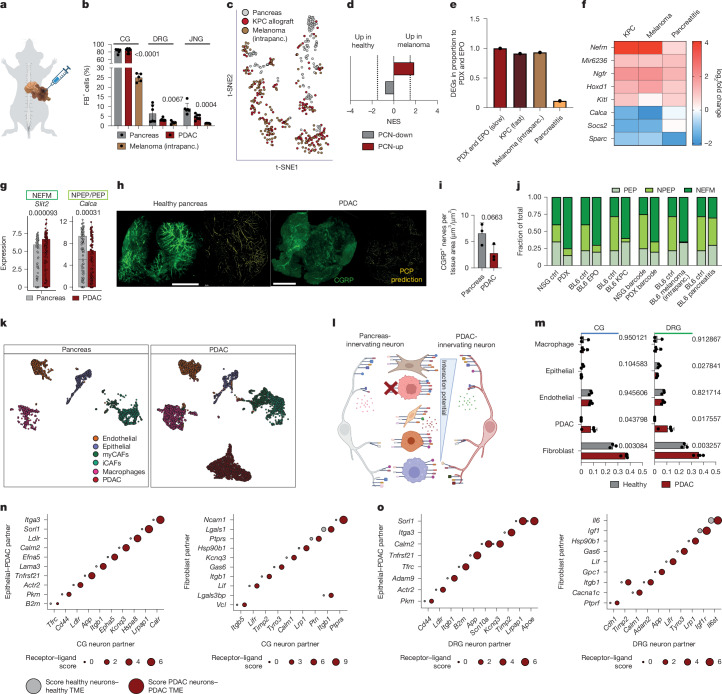
PDAC alters neuronal interactions and attracts specific subtypes in the TME. **a**, Schematic: intrapancreatic melanoma. **b**, Percentage of FB^+^ cells per ganglion in CG, DRG and JNG in healthy pancreas (*n* = 6 mice), PDAC (*n* = 7 mice) and a model of intrapancreatic melanoma (*n* = 5 mice). Two-tailed unpaired *t*-test of PDAC versus melanoma. Mean ± s.d. **c**, *t*-SNE of pancreas, KPC and intrapancreatic melanoma neurons. **d**, Enrichment analysis of PCN-up and PCN-down signatures of DRG neurons in the intrapancreatic melanoma model. **e**, Number of genes with a fold change of > 0.75 compared with controls, between KPC, melanoma and pancreatitis models in relation to PDX and EPO. **f**, Heat map of upregulated and downregulated genes in KPC, melanoma and pancreatitis compared with their respective healthy controls. **g**, Relative gene expression of *Slit2* and *Calca* in pancreas and PDAC (PDX and EPO) DRG neurons. Box plots show median, 25% and 75% quantiles, whiskers are 1.5× interquartile ranges (*n* = 333 pancreas and 605 tumour neurons). **h**, Representative original LSFM images of whole pancreas and PDAC specimen stained with CGRP and transformation. Scale bars, 3,000 μm. **i**, Quantification of CGRP^+^ nerve fibres per tissue area between pancreas (*n* = 3) and PDAC (PDX *n* = 3) specimens. Two-tailed unpaired *t*-test. Mean ± s.d. **j**, DRG subtype composition^26^ of pancreas and tumour (PDX, EPO, KPC, PDX Barcode-seq, melanoma) and pancreatitis-innervating neurons. **k**, *t*-SNE plot of 10X Genomics-based scRNA-seq of pancreas (*n* = 5,700 cells, 3 replicates) or PDX PDAC tumours (*n* = 9,151 cells, 4 replicates), coloured by cell type. CAFs are separated into myofibroblast CAFs (myCAFs) and inflammatory CAFs (iCAFs). **l**, Schematic of receptor–ligand prediction. **m**, Predicted interaction potential. Red, interaction of TME cells (*n* = 16,450 cells, 4 replicates) with PDAC neurons; grey, pancreatic cells (*n* = 20,964 cells, 3 replicates) with pancreas neurons. Multiple unpaired *t*-test. Mean ± s.d. **n**,**o**, Interaction scores of the ten most-changed receptor–ligand interactions between healthy pancreas CG (**n**) or DRG (**o**) neurons and cell types in the healthy pancreas (epithelial and fibroblast) and PDAC CG (**n**) or DRG (**o**) neurons and cell types in the TME (PDAC and CAFs). Source Data

**Extended Data Fig. 11:**
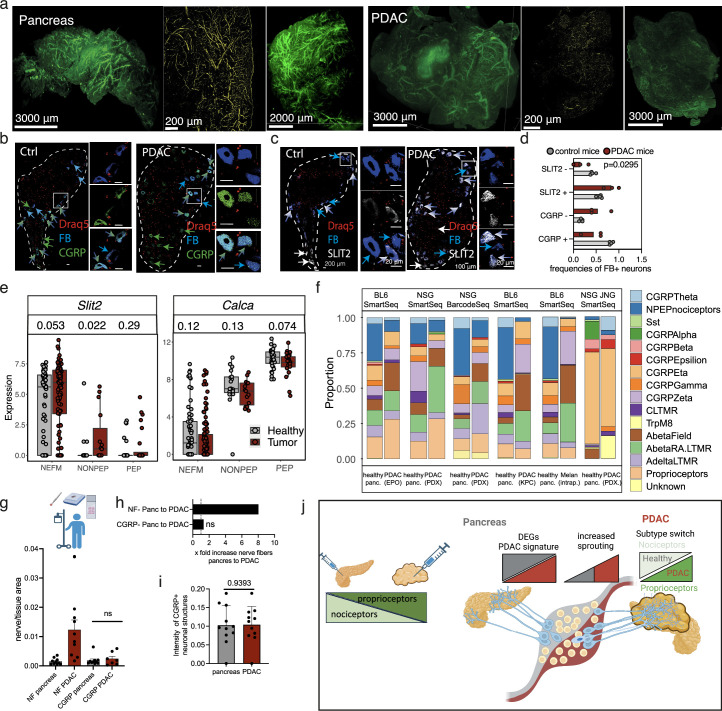
SLIT2-expressing NEFM neurons preferentially innervate cancer. **a**, Representative LSFM staining of CGRP^+^ neurons (green) in the pancreas and PDAC and PCP based prediction (yellow) for quantification. **b**, IF staining of full DRG ganglia section retrogradely labelled (FB, blue) form healthy mice stained with DRAQ5 (red) and antibody for CGRP (*Calca* protein, green) and retrogradely labelled from PDAC mice including zoom-in. **c**, IF staining of full DRG ganglia section retrogradely labelled (FB, blue) form healthy mice stained with DRAQ5 (red) and antibody for SLIT (white) and retrogradely labelled from PDAC mice including zoom-in. **d**, Quantification of FB^+^, SLIT2 or CGRP labelled neurons in DRG n = 3 mice. Two-way ANOVA test. **e**, Gene expression of *Slit2* and *Calca* between pancreas and PDAC neuron for the different DRG subtypes. Box plots in this figure show median, 25%- and 75%-quantiles, whiskers 1.5 interquartile ranges and represent data from 333 (pancreas) and 605 (tumour) cells. Wilcoxon test. **f**, Neuronal subtype proportion of sensory DRG neurons in pancreas versus in PDAC (EPO, PDX, PDX Barcode-seq, KPC) melanoma and in the JNG annotated in a previous report^27^. **g**, Quantification of nerve/tissue area of human pancreas (grey) and treatment naive PDAC (red) stained with NF (n = 10) and CGRP (n = 9 pancreas, n = 7 PDAC). *P* value was determined with two-tailed unpaired t-test. Mean +/− SD. **h**, x-fold increase of NF+ neurons/tissue area or CGRP+ neurons/tissue area from pancreas and PDAC. *P* value was determined with unpaired t-test. Mean +/− SD. **i**, Intensity of CGRP expression within nerve structures in human pancreas (grey) and treatment naive PDAC (red) (n = 10) and CGRP. *P* value was determined with unpaired t-test. Mean +/− SD. **j**, Schematic summary of induced neuronal changes by PDAC. Source Data

To investigate neuronal changes in other cancer models, we injected a melanoma cell line into the pancreas (Fig. 3a). Although we observed similar tracing frequencies between the pancreas and PDAC PDX models, melanoma showed lower innervation by CG (25% FB^+^), DRG (1.7% FB^+^) and JNG (1.3% FB^+^) neurons (Fig. 3b). We next generated Trace-n-Seq data from melanoma-, KPC- and pancreas-innervating neurons (Fig. 3c). In melanoma neurons, PCN-up was enriched, whereas PCN-down enrichment just trended towards control DRG neurons, potentially owing to fast growth with no enrichment in sympathetic neurons (Fig. 3d and Extended Data Fig. 10i). In melanoma DRG neurons, we found depletion of metabolic gene sets similar to that seen in PDAC PDX, but—different from PDAC—enrichment for IL-6 signalling and amino acid transport (Extended Data Fig. 10j). To compare changes induced by cancer and by acute inflammation, we generated Trace-n-Seq data in for 232 healthy neurons and 547 neurons in which pancreatitis was induced by cerulein (Extended Data Fig. 10k–q). Most DEGs in pancreatitis were detected in CG and NEFM neurons. Stress signatures were enriched in NEFM and PEP but not in NPEP or CG neurons, implying subtype-specific changes in pancreatitis. *Trpa1*, a pain-chemosensor, was upregulated in pancreatitis, as reported, but this change was specific to pain-sensing PEP neurons^41^ (Extended Data Fig. 10n). Similar numbers of DEGs were found between healthy and all cancer-infiltrating DRG neurons, but fewer DEGs were found in pancreatitis (Fig. 3e and Extended Data Fig. 10r–t). Some genes, such as *Mir6236*, and those involved in NGF signalling, including *Ngfr* and *Hoxd1*, were upregulated in all three settings^17,42^. In cancer-innervating, but not in pancreatitis DRG neurons, *Nefm* was specifically upregulated, whereas the PEP and NPEP markers *Calca* and *Socs2* were downregulated (Fig. 3f and Extended Data Fig. 10t). PDAC DRG neurons overexpressed the NEFM marker *Slit2*, whereas *Calca* was decreased (Fig. 3g). We also detected fewer (2.3×) CGRP^+^ neurons in 3D images of PDAC, compared with 3D images of healthy pancreas, indicating either preferential attraction of CGRP^neg^ neurons or lower levels of CGRP in PDAC neurons (Fig. 3h–i and Extended Data Fig. 11a). Immunofluorescence microscopy of FB^+^-traced DRGs revealed fewer CGRP^+^ and more SLIT2^+^ neurons in PDAC than in pancreas (Extended Data Fig. 11b–d). However, the expression of *Slit2* and *Calca* in DRG subtypes was similar (NEFM, PEP and NPEP) (Extended Data Fig. 11e). To investigate this, we quantified the frequencies of cancer-innervating DRG subtypes in the six models and respective controls: the EPO model (model 1); both PDX models (using Smart-seq (models 2 and 3) and Barcode-seq (model 4)); the KPC model (model 5); and the intrapancreatic melanoma model (model 6). Across cancer models, we consistently observed increased NEFM but decreased NPEP and PEP neurons, and this was further validated using annotations of an additional atlas^27^. This subtype switch was tumour-specific, and was absent in pancreatitis (Fig. 3j and Extended Data Fig. 11f). In agreement, immunohistochemistry (IHC) of human PDAC and pancreas showed a similar CGRP intensity (expression) but a reduced area of CGRP neurons in PDAC, although the total neuronal area increased (Extended Data Fig. 11g–j). In summary, various models of intrapancreatic cancer, but not pancreatitis, were preferentially innervated by NEFM-type neurons.

## Interactomes of PDAC-innervating neurons

**Extended Data Fig. 12:**
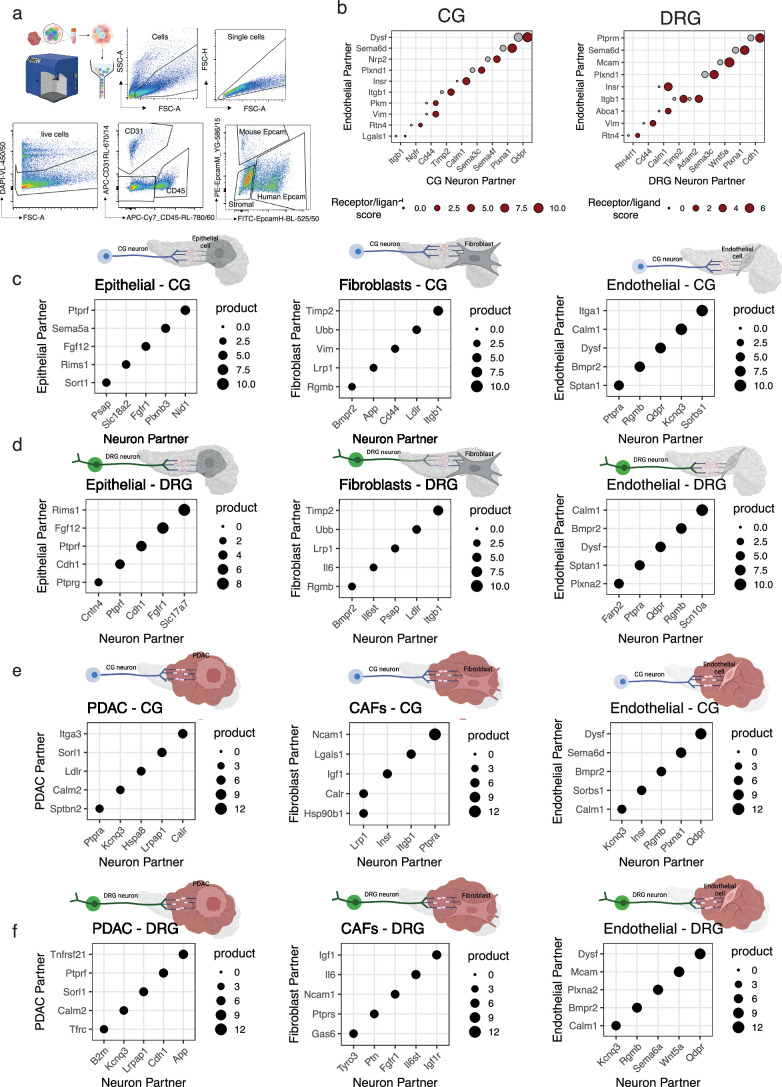
Receptor–ligand pairs overexpressed in PDAC and its TME. **a**, FACS scheme used to identify different TME population in pancreas, PDAC and PDAC denervated samples. **b**, Interaction score of the 10 most-changed receptor/ligand interactions between healthy pancreas CG neurons and endothelial cells in the healthy pancreas (grey) and PDAC CG neurons and endothelial cells in the TME (red). **c**, Interaction score of the 5 strongest receptor/ligand interactions in healthy pancreas CG neurons and cell types in the healthy pancreas (epithelial, fibroblast, endothelial). **d**, Interaction score of the 5 strongest receptor/ligand interactions in healthy pancreas DRG neurons and cell types in the healthy pancreas (epithelial, fibroblast, endothelial). **e**, Interaction score of the 5 strongest receptor/ligand interactions in PDAC CG neurons and cell types in the TME (PDAC, CAFs, endothelial). **f**, Interaction score of the 5 strongest receptor/ligand interactions in PDAC DRG neurons and cell types in the TME (PDAC, CAFs, endothelial).

PDAC is characterized by desmoplastic stroma. To identify neuronal interaction partners in PDAC or pancreas stroma, we bioinformatically integrated our neuronal transcriptome with scRNA-seq data from our PDX model and healthy pancreas (Fig. 3k, Extended Data Fig. 12a and Methods). In the latter, we identified the following cell types: PDAC, healthy epithelial, endothelial, immune and two fibroblast populations (Fig. 3k). To map the neuronal interaction partners, we scored co-expressed ligand–receptor pairs between PDAC neurons and all other PDAC cell types, compared with pancreas neurons and pancreas and its stromal cells^43^ (Fig. 3l,m). Fibroblasts were the strongest neuronal interaction partners in both pancreas and PDAC, but cancer-associated fibroblasts (CAFs) scored higher than healthy ones. Interaction scores remained similar for other TME cell types, but PDAC cells scored higher than healthy epithelial cells with both CG and DRG neurons (Fig. 3m). Next, we ranked the most significantly co-expressed and altered receptor–ligand pairs among all cell types in PDAC compared with pancreas for both CG and DRG neurons. The resulting PDAC– and pancreas–neuron interactomes revealed numerous robustly enhanced receptor–ligand interactions in PDAC, including *Efna5* (PDAC)–*Epha5* (CG); *Gas6* (CAF or endothelial)–*Tyro3* (DRG) or *Il6* (CAF or PDAC)–*Il6st* (DRG). Furthermore, endothelial–neuron interactions through several semaphorins were increased in PDAC, underscoring the crucial roles of these growth factors^44^ (Fig. 3n,o and Extended Data Fig. 12). In summary, we detected a robust enhancement of neuronal interactions in PDAC and its TME, with CAFs as a dominant target. This included a considerable number of known interactions, but also some unexpected ones.

**Extended Data Fig. 13:**
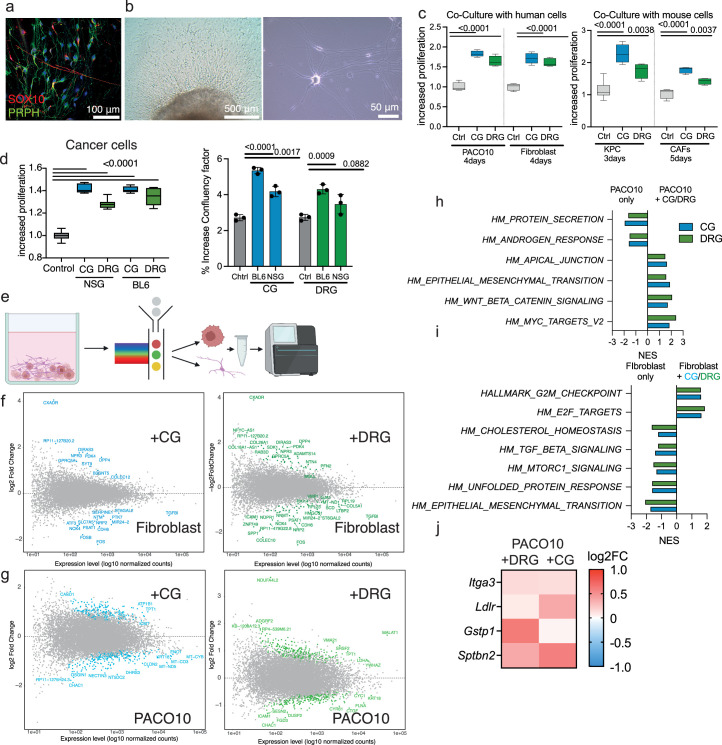
In vitro neuronal co-cultures increase PDAC and CAF proliferation. **a**, IF labelling using PRPH (green) and SOX10 (red, glia marker) of mouse ganglia in vitro to validate neuronal content in culture (performed for n = 6 CG (mice) and 8 DRG (from 3 mice) in 3 experiments). **b**, Representative brightfield microscopy image of in vitro cultured ganglia neurons (from n = 6 mice). **c**, Proliferation of indicated cultured cells in a transwell either alone or with ganglion explants (each ganglion replicate is one mouse): PDAC (PACO10) cells or human fibroblasts alone (ctrl, grey) or cultured in CG (blue)/DRG (green) (n = 8 each, from 2 independent experiments). And KPC cells alone (n = 8) or mouse CAFs alone (n = 4, ctrl, grey) or cultured with CG (blue)/DRG (green) (n = 4 each) analysed by CTB. Ordinary one-way ANOVA. Median +/− SD is shown, **d**, CTB assays of PDAC (PACO) cells cultured in transwells either alone (Ctrl n = 24) or with CG/DRG cultures of ganglia extracted from either NSG or BL6 mice (n = 12). Derived from 3 experiments. Min-max is shown. *P* value was determined by multiple t-test. Every condition was compared to control PACO cells. *P* value < 0.0001 for all conditions; and (2) confluence measurement between control PDAC (PACO43) cells or PDAC cells supplemented with conditioned medium from ganglion explants for 4 days of CG/DRG from NSG and BL6 mice. The increase in confluency was measured with the cytosmart software at day 0 and after 3 days of co-culture (n = 3) Experiment was repeated 3 times. *P* value was determined by multiple t-test. Mean +/− SD. **e**, Schematic outline of co-culture of in vitro ganglion with PDAC (PACO10)/Fibroblast cells, FACS separation and bulk RNA-seq approach. **f**, Volcano Plot of Fibroblasts control vs CG (blue) or DRG (green) co-cultured fibroblast cells (n = 3 replicates/mice). Genes are considered to be significantly differentially expressed at an adjusted *P* value of <0.1 (DESeq Wald test, Benjamini–Hochberg correction for multiple testing). **g**, Volcano plot of PDAC cells control vs CG (blue) or DRG (green) co-cultured PDAC cells (n = 3 replicates/mice). Genes are considered to be significantly differentially expressed at an adjusted *P* value of <0.1 (DESeq Wald test, Benjamini–Hochberg correction for multiple testing). **h**, GSEA of PDAC cells cultured alone vs. with CG (blue) and DRG (green) cells. **i**, GSEA of fibroblast cells co-cultured with CG (blue) and DRG (green) cells. **j**, Heat map of DEGs in vitro in the co-culture of PACO10 cells with CG/DRG cells also found differentially expressed in PDX PDAC cells between control and denervated tumours in vivo. Source Data

To validate the predicted cell–cell interactions, we co-cultured neuronal explants with human or mouse PDAC cells, human fibroblasts or CAFs, or exposed PDAC cells to neuron-conditioned medium (NSG and BL6). Neurons or neuron-conditioned medium increased (30–50%) proliferation in all settings (tumour, fibroblast and CAF) and this effect was strongest in CG co-cultures or conditioned medium (Extended Data Fig. 13a–d). RNA-seq analysis of co-cultured PDAC cells and fibroblasts or CAFs revealed a neuron-mediated upregulation of proliferation (MYC targets, G2M checkpoint) (Extended Data Fig. 13e–j). Collectively, these data identify neuron-mediated pathways that directly affect the proliferation of PDAC, fibroblasts and CAFs. However, molecular changes detected in vivo were only reproduced for a subset of factors in vitro, highlighting the importance of in vivo models to dissect the complex physiology of neuron-driven tumour ecosystems (Extended Data Fig. 13j).

## Denervation sensitizes PDAC to immune therapy

**Fig. 4:**
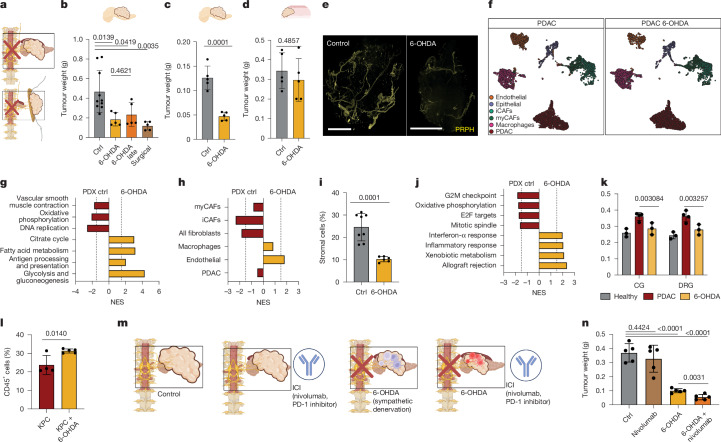
Denervation slows PDAC growth and induces pro-inflammatory changes in CAFs, sensitizing PDAC to ICIs. **a**, Schematic of sympathetic denervation in orthotopic and subcutaneous tumour. **b**, Tumour weight of orthotopic PDX mice; control (*n* = 10 mice) versus different denervation conditions (n = 5 mice each). Two-tailed unpaired-*t*est. Mean ± s.d. **c**, Tumour weight of orthotopic KPC-allograft mice; control versus the 6-OHDA denervation condition (*n* = 5 mice). Two-tailed unpaired *t*-test. Mean ± s.d. **d**, Tumour weights of subcutaneously transplanted PDAC PDX mice; control versus the 6-OHDA denervation condition (*n* = 5 mice). Two-tailed unpaired *t*-test. Mean ± s.d. **e**, Representative PRPH-stained LSFM imaging of control PDAC or denervated tumours. Scale bars, 1,000 μm. **f**, *t*-SNE plot of 10X Genomics-based scRNA-seq of PDX PDAC tumours (*n* = 9,151 cells, 4 replicates) or denervated PDX PDAC tumours (*n* = 9,607 cells, 3 replicates), coloured by cell type. **g**, GSEA of PDAC cells for control versus denervated PDX tumours. **h**, GSEA of G2M checkpoint for control versus denervated tumours. **i**, Relative percentage of stromal cells in control and denervated PDX tumours, analysed by flow cytometry. Two-tailed unpaired *t*-test. Mean ± s.d. **j**, GSEA of fibroblasts from control versus denervated PDX tumours. **k**, Predicted interaction potential by fibroblast and ganglion type (grey, healthy pancreas fibroblasts with pancreas neurons (*n* = 3 mice); red, PDAC fibroblasts with PDAC neurons (*n* = 4 mice); yellow, denervated PDAC fibroblasts with PDAC neurons (*n* = 3 mice)). Two-tailed unpaired *t*-test. Mean ± s.d. **l**, Percentage of CD45^+^ cells in control (*n* = 4 mice) and denervated KPC tumours (n = 5 mice) analysed by flow cytometry. Two-tailed unpaired *t*-test. Mean ± s.d. **m**, Schematic of treatment regimen. **n**, Tumour weight of KPC-allograft mice; control versus nivolumab, 6-OHDA and combinational treatment (nivolumab + 6-OHDA) (*n* = 5 mice). Two-tailed unpaired *t*-test. Mean ± s.d. Source Data

**Fig. 5:**
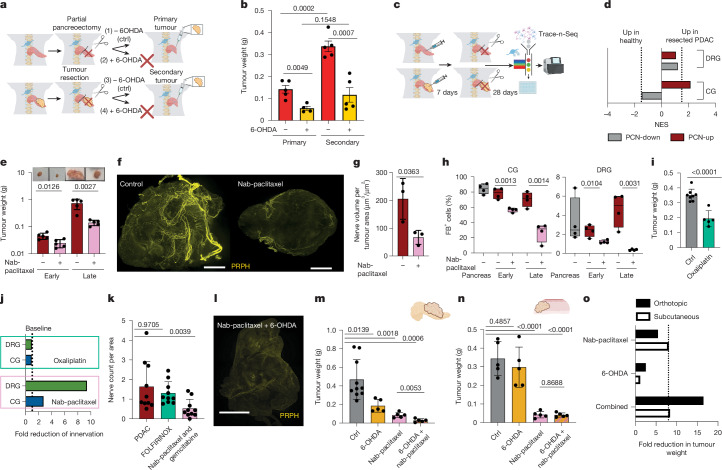
Nab-paclitaxel reduces tumour growth by depleting tumour-infiltrating neurons. **a**, Experimental set-up. **b**, Tumour weight of primary orthotopic (1) untreated (*n* = 5), (2) 6-OHDA denervated (*n* = 4), sham-operated (partial pancreatomy) and (3) secondary untreated (*n* = 5) or (4) 6-OHDA denervated, (primary tumour resected) PDX mice (*n* = 5). Two-tailed unpaired *t*-test. Mean ± s.e.m. **c**, Experimental set-up: Trace-n-Seq and PDAC resection. **d**, Enrichment analysis of PCN-up and PCN-down signatures of CG and DRG neurons 28 days after tumour resection (tumour-free) or sham operation (partial pancreatomy) in a comparison of pancreas and PDAC neurons. **e**, Representative images and tumour weight of mice treated with nab-paclitaxel (10 mg kg^−1^) compared with untreated controls after two cycles (early) and four cycles (late) (*n* = 5). Two-tailed unpaired *t*-test. Mean ± s.d. **f**, Representative LSFM images of PDAC control versus nab-paclitaxel-treated mice. Scale bars, 1,000 μm. **g**, Quantification of nerve volume per tumour area between PDAC control (*n* = 3) and nab-paclitaxel-treated (*n* = 3) specimens. Two-tailed unpaired *t*-test. Mean ± s.d. **h**, FB^+^ CG and DRG cells after retrograde tracing of pancreas (grey), PDX control (red), or after treatment with nab-paclitaxel (pink) for two or four cycles. Two-tailed unpaired *t*-test, *n* = 4 mice per condition. **i**, Tumour weight of control mice or mice treated with oxaliplatin, after two cycles (*n* = 5). Two-tailed unpaired *t*-test. Mean ± s.d. **j**, Relative reduction of FB^+^ cells in CG and DRG after retrograde tracing of PDX; control or after treatment with nab-paclitaxel or oxaliplatin (*n* = 4). **k**, Quantification of nerves per tissue area of human PDAC (naive or after neoadjuvant FOLFIRINOX or nab-paclitaxel and gemcitabine treatment) from neurofilament-stained IHC slides (*n* = 10 patients each). Mann–Whitney test. Mean ± s.e.m. **l**, Representative LSFM images after treatment with nab-paclitaxel and 6-OHDA. Scale bar, 1,000 μm. **m**, Tumour weight of orthotopic PDX mice (control or treated with 6-OHDA, nab-paclitaxel (10 mg kg^−1^) or both) (*n* = 5). Two-tailed unpaired *t*-test. Mean ± s.d. **n**, Tumour weight of subcutaneous PDX mice (control or treated with 6-OHDA, nab-paclitaxel (10 mg kg^−1^) or both) (*n* = 5). Two-tailed unpaired *t*-test. Mean ± s.d. **o**, Relative reduction in tumour weight after various treatments in the orthotopic and subcutaneous models. Source Data

**Extended Data Fig. 14:**
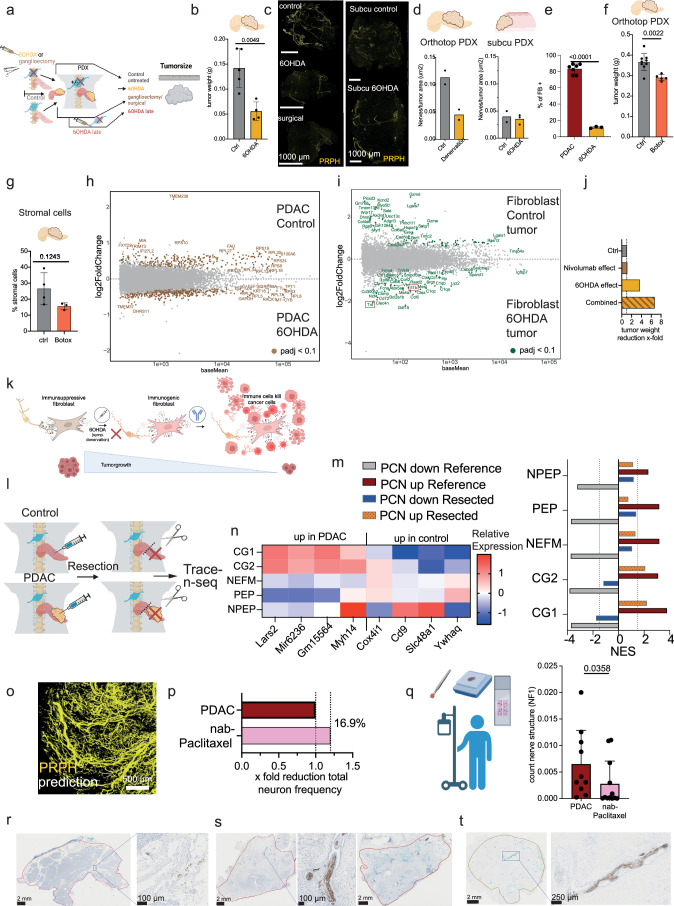
Denervation inhibits tumour growth and local relapse in PDAC. **a**, Schematic of in vivo denervation approaches. **b**, Tumour weight of orthotopic PDX mice, control (n = 5) vs. 6-OHDA denervation mice (n = 5) at an early time point (tumour establishment). *P* value was determined by two-sided unpaired t-test. Mean +/− SD is shown. **c**, Representative pictures of LSFM imaging of orthotopic and subcutaneous tumours of untreated control PDAC or denervated tumours (surgical or 6-OHDA) stained with an anti-PRPH antibody (yellow). **d**, PCP based quantification of PRPH^+^ nerve fibres/tissue area between PDAC controls (n = 2) and denervated PDAC specimen (n = 2) and between subcutaneous PDAC controls (n = 2) and denervated PDAC-s.c. (n = 3). **e**, Validation of denervation efficacy: % of FB+ cells in the CG traced from untreated PDAC control tumours and 6-OHDA denervated tumours (n = 7/3). *P* value was determined by two-tailed unpaired t-test. Mean +/− SD is shown. **f**, Tumour weight of orthotopic PDX mice, control (n = 9) vs. Botox (n = 5) denervation. *P* value was determined by two-tailed unpaired t-test. Mean +/− SD is shown. **g**, Relative percentage of stroma cells in control (n = 4) and BOTOX denervated PDX tumours (n = 3) analysed by flow cytometry. Two-tailed unpaired t-test. Mean +/− SD is shown. **h**, Volcano plot of DEGs from PDAC cells of control (n = 4 mice) vs. denervated (n = 3 mice) PDX tumours. Genes are considered to be significantly differentially expressed at an adjusted *P* value of <0.1 (DESeq Wald test, Benjamini–Hochberg correction for multiple testing). **i**, Volcano plot of Fibroblast cells from control (n = 4 mice) vs denervated (n = 3 mice) PDX tumours. Genes are considered to be significantly differentially expressed at an adjusted *P* value of <0.1 (DESeq Wald test, Benjamini–Hochberg correction for multiple testing). **j**, X-fold reduction of tumour weight in mice with 1. Control tumours treated with vehicle control, 2. Tumours treated with ICI nivolumab mono, 3. Denervation via 6-OHDA mono, 4. Combination of 6-OHDA based denervation and Nivolumab treatment. **k**, Schematic of immune phenotype in fibroblast upon denervation and enablement of ICI-based tumour killing. **l**, Schematic: PDAC/Pancreas resection after FB injection. **m**, PCN-signature enrichment in neuronal subtypes compared to the PDX mice. **n**, Heat map of DEGs from the PCN- signature in the resected model. **o**, Representative image of PCP based quantification of tumours. **p**, x-fold reduction of total neurons per CG (NeuO+ cells) after nab-paclitaxel (pink) treatment (4 cycles) in comparison to untreated control tumours (red). Changes were ns (two-tailed unpaired t-test, n = 3). **q**, Quantification of count of nerve structures of human PDAC naive (red), and PDAC after neoadjuvant nab-paclitaxel/Gemcitabine (pink) treatment of neurofilament-stained IHC slides (n = 10). P value was determined by Mann–Whitney test.+/− SD. **r**, Representative images of NF1 stained IHC slides of human pancreas, **s**, PDAC naive, **t**, and PDAC post nab-paclitaxel treatment. Source Data

To examine the role of neurons in PDAC growth in vivo, we depleted sympathetic neurons through surgical celiac ganglionectomy or using the anti-catecholaminergic neurotoxin (6-OHDA) in PDX and KPC models. Both methods reduced tumour weight by up to threefold, even when neurons were ablated 3–4 weeks after tumour establishment (Fig. 4a–c and Extended Data Fig. 14a,b). Denervation was confirmed by 3D imaging of PRPH-stained tumours and pancreas CG FB^+^ tracing efficiency was reduced eightfold after 6-OHDA treatment (Fig. 4e and Extended Data Fig. 14c–e). An identical experiment in poorly innervated subcutaneous PDAC xenografts reduced neither tumour size nor neuronal infiltration, excluding neuron-independent anti-cancer effects of 6-OHDA (Fig. 5d and Extended Data Fig. 14c,d). Although inferior to 6-OHDA, intratumoral injection of Botox also reduced tumour size (20.8%) (Extended Data Fig. 14f). In scRNA-seq data for PDAC after 6-OHDA denervation, gene sets associated with proliferation and oxidative phosphorylation were depleted in PDAC cells and CAFs, whereas inflammation and citrate-cycle signatures were enriched compared with control tumours (Fig. 4f–h). In line with this, we detected a reduction of CAFs after 6-OHDA treatment, and to a lesser degree after Botox treatment (Fig. 4h,i and Extended Data Fig. 14g). In addition, after denervation, the interaction scores of CAFs with CG and DRG neurons dropped back to that of healthy fibroblasts. These denervated CAFs upregulated the molecular programs ‘interferon-α response’ and ‘allograft rejection’ and genes such as *Tnf* or *Il1b*, suggesting a pro-inflammatory state (Fig. 4j,k and Extended Data Fig. 14h–i). In support of a pro-inflammatory PDAC TME, immune-competent KPC tumours contained more CD45^+^ cells after 6-OHDA treatment (Fig. 4l). To investigate whether PDAC neurons suppress anti-tumour immunity through CAFs, we combined 6-OHDA with the immune-checkpoint inhibitor (ICI) nivolumab. Alone, ICI did not affect KPC tumour size, but in combination with 6-OHDA, it reduced tumour size by 5.7-fold, suggesting that denervation increases ICI efficiency (Fig. 5m,n and Extended Data Fig. 14j). These findings align with reports that targeting neurons enhances immunotherapies in other cancers, and could open new treatment options for PDAC, an otherwise immunologically ‘cold’ cancer (Extended Data Fig. 14k).

## CG reprogramming promotes local relapse

Local relapse is a major problem after PDAC resection. To assess the neuronal contribution to this, we studied four groups of mice: two were injected with PDX cell lines and two received sham injections (PBS + Matrigel intrapancreatic). After 10 weeks, they underwent either tumour resection (PDX) or partial pancreatectomy (sham). Subsequently, new PDAC cells were injected into the remaining pancreas of all four groups and one group from each arm either received 6-OHDA or control NaCl injections. This revealed that, first, secondary PDAC tumours in mice that previously carried (resected) tumours were 2.5-fold larger; second, 6-OHDA reduced tumour growth in primary and secondary conditions; and, third, 6-OHDA-mediated denervation prevented increased growth of secondary tumours (Fig. 5a,b). These data show that neurons are key drivers for a ‘primed TME’ that leads to accelerated secondary tumour outgrowth, which could be targeted by denervation. To uncover a persisting molecular footprint of sensitized neurons beyond resection, we injected FB into pancreas or small tumours. Seven days later, we either resected tumours or performed partial pancreatectomy (control), and removed ganglia after 28 days to perform Trace-n-Seq (Fig. 5c and Extended Data Fig. 14l). Compared with neurons from pancreatectomized (control) mice, PDX CG neurons maintained overexpression of genes such as *Mir6236* even at 4 weeks after resection. Moreover, PCN-up and PCN-down signatures were enriched in CG neurons, which suggests that sympathetic neurons maintain a ‘cancer nerve state’ (Fig. 5c,d and Extended Data Fig. 14l–m). In summary, tumour-reprogrammed sympathetic nerves maintain tumour-promoting features well beyond tumour resection and drive local recurrence of PDAC.

## Nab-paclitaxel denervates PDAC

**Extended Data Fig. 15:**
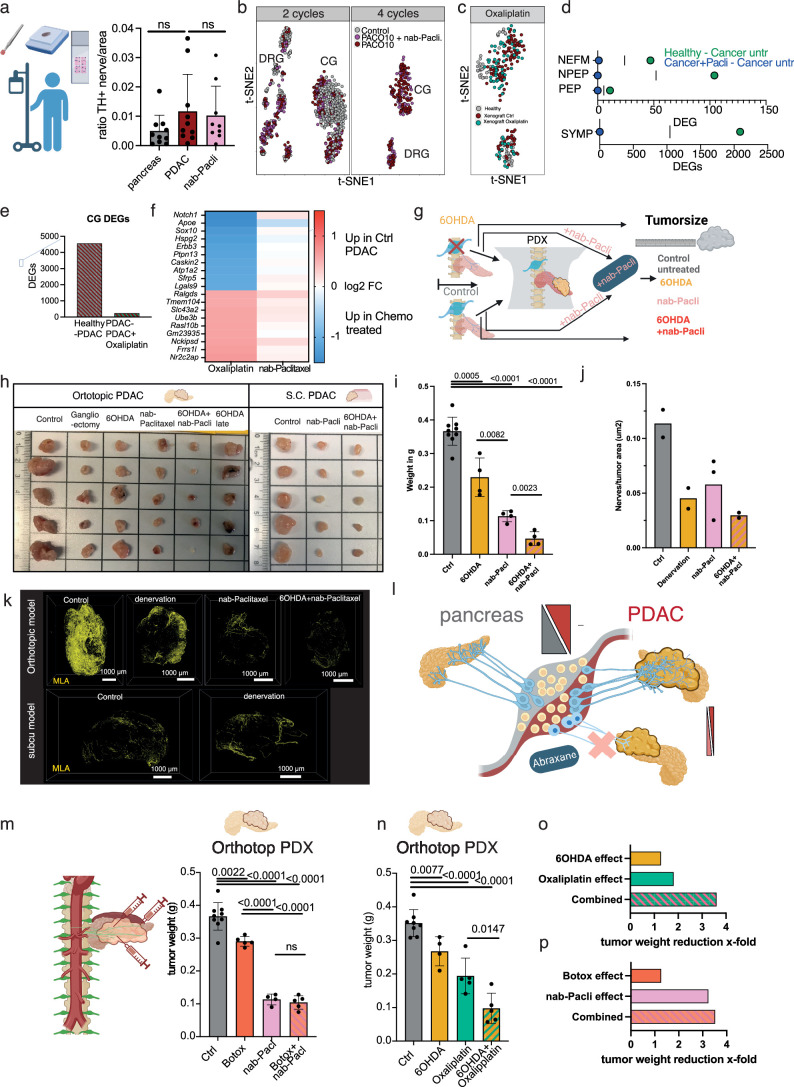
Denervation synergizes with taxane-based chemotherapy in PDAC. **a**, Quantification of TH + nerve/tissue area of human pancreas (grey, n = 10), PDAC naive (red, n = 10), and PDAC after neoadjuvant nab-paclitaxel/Gemcitabine (pink, n = 9) treatment of TH stained IHC slides (n = 10). P value was determined by two-tailed unpaired t-test +/− SD. **b**, t-SNE of retrogradely labelled CG and DRG neuronal cells traced from healthy pancreas, PDX control and nab-paclitaxel treated PDX mice after 2 and 4 cycles (n = 4 mice per condition). **c**, t-SNE of retrogradely labelled CG neuronal cells traced from healthy pancreas, PDX control and Oxaliplatin-treated PDX mice (n = 3 mice per condition). **d**, Number of DEGs in CG and DRG neuronal subtypes between healthy pancreas and PDAC neurons, PDAC control and PDAC neurons of nab-paclitaxel treated mice. **e**, Number of DEGs in CG neuronal cells between healthy pancreas and PDAC neurons, PDAC control and PDAC neurons of Oxaliplatin-treated mice. **f**, Heat map of DEGs in CG neuronal cells between deregulated between healthy pancreas and PDAC neurons and PDAC control and PDAC neurons of nab-paclitaxel and Oxaliplatin-treated mice. **g**, Schematic of in vivo denervation and combinatorial treatment approaches. **h**, Images of extracted tumours after different denervation and treatments in the orthotopic and subcutaneous PDX model. N = 5. **i**, Tumour weight of orthotopic control (n = 9), 6-OHDA denervated (n = 4), nab-paclitaxel (10 mg/kg) (n = 4) and the combination of treated PDX mice (n = 4) after reduced cumulative doses. *P* value was determined by two-tailed unpaired t-test. Mean +/− SD is shown. **j**, PCP based quantification of PRPH^+^ nerve fibres/tissue area between PDAC controls (n = 2) and denervated PDAC specimen (n = 2) and between subcutaneous PDAC controls (n = 2) and denervated PDAC-*s.c*. (n = 3). *P* value was determined by unpaired t-test. Mean +/− SD is shown. **k**, Representative images of the PCP based prediction of innervation in orthotopic PDAC specimen of control and after treatment (denervation, nab-paclitaxel, 6-OHDA+nab-paclitaxel) and representative images of the PCP based prediction of innervation in subcutaneous PDAC specimen of control and after denervation. **l**, Schematic summary of results. **m**, Schematic and tumour weight of orthotopic control (n = 9), Botox denervated (n = 5), nab-paclitaxel (10 mg/kg) (n = 4) and the combination of treated PDX mice (n = 5). P value was determined by two-tailed unpaired t-test. Mean +/− SD is shown. **n**, Tumour weight of orthotopic control (n = 8), 6-OHDA denervated (n = 4), Oxaliplatin (n = 5) and the combination of treated PDX mice (n = 5). P value was determined by two-tailed unpaired t-test. Mean +/− SD is shown. **o**, Relative tumour weight reduction upon different treatments (6-OHDA, Oxaliplatin, Combi) in the orthotopic model in comparison to control mice. **p**, Relative tumour weight reduction upon different treatments (Botox, nab-paclitaxel, Combi) in the orthotopic model in comparison to control mice. Source Data

Combined with gemcitabine, the taxane nab-paclitaxel is a standard treatment regimen for PDAC. By blocking microtubules, nab-paclitaxel inhibits mitotic spindles, inducing cell-cycle arrest, and affects axonal transport that depends on microtubules—preferentially sensory neurons. The resulting peripheral neuropathy is a common adverse effect of taxanes, leading to sensory dysaesthesia and discontinuation of treatment. However, severe neuropathy is associated with better outcomes for PDAC treated with gemcitabine and nab-paclitaxel^45^. Therefore, we investigated the effect of nab-paclitaxel on PDAC neurons with Trace-n-Seq. Nab-paclitaxel reduced tumour size, as expected, but also decreased intratumoral axonal sprouting detected by 3D LSFM. After treatment, neurons per CG remained stable, whereas neuronal fibres within tumours were reduced by 62% (Fig. 5e–g and Extended Data Fig. 14o,p). In addition, FB^+^ CG PDAC neurons decreased continuously over four treatment cycles from 84% to 27%. FB^+^ DRG neurons decreased by twofold after two and by 9.8-fold after four cycles (Fig. 5h). Therefore, nab-paclitaxel disrupts intratumoral neuronal sprouting to the point that almost no sensory neurons project into the tumour. Another standard PDAC regimen, FOLFIRINOX, also induces neuropathies through oxaliplatin by calcium depletion and sodium-channel depolarization. However, in oxaliplatin-treated PDX mice, the size of tumours was reduced, but the number of FB^+^ PDAC neurons was not^46^ (Fig. 5i,j). In sections from patient samples, reduced nerve/tumour ratios and neuronal structures were detected in patients treated with gemcitabine and nab-paclitaxel, but not in those treated with FOLFIRINOX, validating that taxanes specifically reduce neuronal infiltration in PDAC (Fig. 5k and Extended Data Fig. 14q–t). Furthermore, TH neurons increased in PDAC compared with healthy pancreas, but did not significantly decrease after taxane therapy, suggesting that taxanes preferentially target sensory neurons (Extended Data Fig. 15a). These data show that nab-paclitaxel, but not oxaliplatin, targets intratumoral neuronal sprouting mostly of sensory neurons and thus contributes to the anti-tumour effect of nab-paclitaxel.

## Combinatorial denervation disrupts PDAC

By Trace-n-Seq analysis of pancreas, untreated PDX, oxaliplatin- and nab-paclitaxel-treated PDX tumours after two or four treatment cycles, we detected multiple DEGs between healthy and PDAC neurons (Extended Data Fig. 15b–f). However, neurons that underwent either therapy clustered closely with untreated PDAC neurons (Extended Data Fig. 15d,e). Thus, although nab-paclitaxel causes severe intratumoral neuropathy, which affects DRG-based sensory neurons in particular, persisting PDAC neurons remain transcriptionally stable, suggesting that targeting the remaining tumour neurons could be a therapeutic option.

To target all PDAC neurons, we combined sympathetic neuron ablation by 6-OHDA with nab-paclitaxel, mostly targeting sensory neurons. As single agents, they reduced PDAC growth by 2.5- or 5.5-fold respectively, but combined they reduced tumour size by up to 16.5-fold (Fig. 5m,o and Extended Data Fig. 15g–i). This synergistic effect led to decreased neuronal tumour infiltration (Fig. 5l and Extended Data Fig. 15j,k). The effect of 6-OHDA effect as a single or combined treatment was observed in orthotopic, but not in poorly innervated subcutaneous PDX tumours, demonstrating specificity (Fig. 5n and Extended Data Fig. 15k–l). Combination treatment with Botox did not increase the growth-inhibiting effect of nab-paclitaxel, potentially because both preferentially target sensory neurons (Extended Data Fig. 15m,p). Oxaliplatin with 6-OHDA only led to an additive effect, not a synergistic effect, compared with single-agent treatments (Extended Data Fig. 15n–o). In summary, our data show that the anti-PDAC effects of nab-paclitaxel—and to a lesser extent, oxaliplatin—are considerably enhanced by sympathetic neuron ablation. This strategy warrants evaluation in prospective clinical trials.

## Discussion

With our introduction of Trace-n-Seq technology, neurons innervating any organ or healthy or cancerous tissue in animal models can now be robustly identified and molecularly characterized at single-cell resolution (see Supplementary Discussion). When applied to the healthy pancreas, this approach revealed marked heterogeneity among neuronal subtypes originating from peripheral ganglia. In PDAC, neurons are reprogrammed by cancer cells, which results in extensive sprouting and a shift to sensory neurons of the NEFM subtype. Increased neural connections to CAFs and tumour cells, as revealed by the establishment of a neuronal interactome, activate pathways that drive tumour progression. We identified a unique PCN expression signature, which persists after tumour resection and might promote tumour proliferation and local relapse. Denervation de-represses inflammatory CAFs, raising the possibility of sensitizing PDAC to immune-checkpoint blockade. Notably, treatment with nab-paclitaxel reduces tumour growth not only by targeting tumour cells, but also by disrupting neuronal infiltration—a novel mechanism of action that oncologists have unknowingly used to targeted cancer nerves for decades. When combined with sympathetic denervation, synergistic effects inhibiting PDAC growth can be achieved, underscoring the importance of the cancer–nerve axis. These findings emphasize the potential of therapeutic strategies that target the complex interactions of PDAC with the peripheral nervous system, which might be applicable to other cancers. The Trace-n-Seq technology will enhance the identification of crucial drug targets in neuron-stimulated carcinomas and beyond.

## Methods

### Ethics

All animal procedures were performed in accordance with German guidelines and were approved by the ethics committee for animal experimentation of Karlsruhe (Regierungspräsidium Karlsruhe; G230/19, G49/19, G105/17 and G148/21). Mouse tumour samples did not exceed the maximum size of 1.5 cm allowed by the ethics committee.

Human tissue samples were obtained and approved by the ethical committee of the University of Heidelberg (case numbers S-206/2011, S-206/2011 and 206/2005) in accordance with the Declaration of Helsinki; written informed consent was obtained from all patients.

### Mouse strains

Wild-type mice had a C57BL/6 (BL6) background (Janvier Labs). NOD.*Prkdc*^*scid*^ *Il2rg*^null^ (NSG) mice were bred and housed under specific-pathogen-free conditions at the central animal facility of the German Cancer Research Center (DKFZ). Female and male mice were used for the studies. Mice older than 8 weeks were used for experiments. Sample sizes were chosen on the basis of previous studies and literature to ensure adequate power to detect biologically relevant differences. Mice were randomized into experimental groups at the start of each experiment when feasible. Blinding was not performed during data collection, but analyses were performed using predefined criteria to minimize bias. All animal experiments were approved by the governmental committee for animal experimentation (Regierungspräsidium Karlsruhe; G230/19, G49/19, G105/17 and G148/21).

### Whole-mount immunostaining and clearing procedure

Intact tumours or pancreata were immunostained and cleared following the iDISCO+ protocol^22^. In brief, mice were euthanized, and pancreas or tumour was resected and washed in 10 ml phosphate-buffered saline (PBS), followed by fixation in 10 ml 4% paraformaldehyde (PFA) in PBS. Dissected tissues were fixed overnight at 4 °C in 4% PFA. Samples were washed in PBS and dehydrated using a graded series of methanol solution (20%, 40%, 60%, 80% and 100% methanol, diluted in PBS) for one hour each at room temperature. Next, samples were washed twice with 100% methanol and then incubated overnight at 4 °C while shaking in 66% dichloromethane (DCM; Sigma 270997; 12 × 100 ml) and 33% methanol at room temperature. Samples were then washed twice to remove DCM. Next, dehydrated tissue was bleached in methanol and 5% hydrogen peroxide overnight at 4 °C. Samples were rehydrated using a graded series of methanol solution, permeabilized in 20% dimethyl sulfoxide (DMSO), 0.16% Triton X and 23 g l^−1^ glycine in PBS for two days at 37 °C and incubated in blocking buffer: PTwH (0.2% Tween-20, 10 mg l^−1^ heparin in PBS), 5% DMSO and 3% bovine serum albumin (BSA) for three days at 37 °C. Tissues were incubated with primary antibodies at 37 °C for 1 week (for antibody list, see Supplementary Table 1), washed in PTwH and incubated with secondary antibodies for two to three days at 37 °C. Samples were washed in PTwH, dehydrated in a graded methanol series and equilibrated in 66% dichloromethane and 33% methanol overnight at room temperature. Methanol was washed off in 100% dichloromethane twice for 15 min. Samples were transferred to dibenzyl ether to complete the clearing. A total of 8 pancreatic samples and 23 tumour samples (orthotopic and subcutaneous) were subjected to tissue clearing. Representative images are provided. For comparative analyses, the sample sizes used are indicated in the corresponding quantification plots. In addition, the combined celiac region, including the pancreas and spleen, as well as the spine, was cleared once to serve as a visual representation.

### LSFM, immunofluorescence and image analysis

Cleared samples were imaged on a light-sheet fluorescent microscope (LaVision Biotech Ultramicroscope II) equipped with an Andor Neo sCMOS camera (Andor) and a LaVision LVMI 4× NA 0.3 objective with a working distance of 6 mm and a correction ring that allows the refractive index to be adjusted in the range 1.30–1.60 (here: DBE, NA: 1.55) (LaVision Biotech). Single mosaic images were first stitched using TeraStitcher v.7.3.1 in ImSpector (Lavision), then converted to Nis-Elements files (.nd2) in the software. Three-dimensional reconstruction of samples was then performed in NIS-Elements (versions 5.21, 5.41 and 5.42 and their respective subversions; Nikon), as well as the associated 3D view and uncompressed movies (.avi), using various render modes (maximum intensity projection, alpha blending, depth-coded alpha blending, alpha blending shading and depth-coded alpha blending with shading). Movies rendered with different modes, and LUTs (lookup tables) were combined in Adobe Premiere Pro CS6 and converted into VBR H.264 format (.mp4) to allow easy viewing on different platforms. Other immunofluorescence images were obtained with a Zeiss LSM 700 using ZEN blue v.2.5 (Zeiss).

### Retrograde labelling of neurons

To retrogradely label pancreas- or PDAC-innervating neurons, adult mice (8–15 weeks) were anaesthetized by isoflurane and application of a subcutaneous carprofen depot 30 min (5 mg kg^−1^) before the surgery. The skin overlying the organs was shaved and a 5-mm incision was made directly on top of the pancreas or the tumour, respectively. The tissue was microdissected and Fast Blue (FB) (Polisciences, 17740-1), reconstituted as a 1% solution in distilled water, was injected slowly at several spots into the pancreas or PDAC of NSG and BL6 mice using a Hamilton (20 µl, 29G) syringe. The injection site was rinsed with 0.5 ml saline (Patterson Veterinary) to wash away any leaking dye before the incision was closed. Mice were euthanized between 5 days and 30 days after injection for histology or scRNA-seq. For retrograde labelling of the colon and spleen, the same operation was performed as described above, but the tracer was slowly injected into the spleen or the colon. For retrograde labelling of peritoneum-innervating neurons, the tracer was further diluted to obtain 50 ml solution and injected intraperitoneally (i.p.).

Mice were euthanized between 5 days and 28 days after tracer injection (depending on the specific experiments; normal tracing period 10 days with sufficient labelling after 5 days; for resection experiments mice were euthanized after 28 days of tracing) for IHC or scRNA-seq.

Similarly, for AAV injections, mice were injected with AAV6 or FluoroRuby (FR). For co-injections, AAV6 or FR were mixed 1:1 with FB and injected into the pancreas. For different organ labelling, FB was injected as described above and FR was injected into the spleen by microinjection. Mice were euthanized after 7–10 days for the FR tracing and at different time points between 5 days and 28 days for AAV6 with sufficient labelling results starting from 28 days.

### Dissection of the CG

The CG is small and difficult to distinguish from the surrounding tissues. Mice were euthanized and overlying organs were removed. Dissection was performed using a stereotactic microscope equipped with a light source. NSG and BL6 mice were used for single-cell experiments and for retrograde tracing from the pancreas, PDAC, peritoneum, colon and spleen. Stainings of the whole ganglia (with and without retrograde tracing) were used for quantification of the proportion of sympathetic cell types. The CG was prepped out and put in artificial cerebrospinal fluid (ACSF) on ice for further use.

### Dissection of the DRG

Mice were euthanized and the spine was removed and cut in the middle. DRGs of interest were collected in freshly oxygenated ice-cold ACSF. Dissection was performed using a stereotactic microscope equipped with a light source. NSG and BL6 mice were used for single-cell experiments and for retrograde tracing from the pancreas, PDAC, peritoneum, colon and spleen. Stainings of the whole ganglia (with and without retrograde tracing) were used for quantification of the proportion of sensory cell types and their soma area. The DRGs in every segment of interest were prepped out and put in ACSF on ice for further use.

### Dissection of the JNG

The JNG is a small, complex structure located adjacent to the vagus nerve, making it challenging to isolate. To access the JNG, the mouse was placed in a supine position. The ventral neck area was carefully opened, and the sternohyoid muscle was retracted. The ganglion, situated adjacent to the carotid artery and jugular vein, was carefully dissected free using fine forceps and micro-scissors under a stereotactic microscope. Once isolated, the JNG was immediately transferred to ice-cold ACSF for further use.

### Dissection of the sympathetic trunk ganglia

After euthanasia, the overlying organs and tissues were removed to expose the spinal region. A midline incision along the back provided access to the thoracic and lumbar vertebrae. The spinal muscles were retracted to reveal the sympathetic trunk ganglia positioned adjacent to the vertebral bodies. The ganglia were carefully dissected free from the connective tissue using fine dissection tools under a stereotactic microscope. The isolated ganglia were placed in ice-cold ACSF for subsequent analysis.

### Sorting of retrogradely labelled neurons

Adult mice with retrogradely labelled neurons were euthanized by cervical dislocation. Th5–Th13 DRGs and CG were quickly removed without nerves attached. Ganglia were immediately digested with a pre-heated (37 °C) digestion mixture.

In brief, 1 ml of digestion solution contains 140 µl TrypLE Express (Life Technologies), 740 μl papain (Worthington, LS003126; 41 mg ml^−1^), 60 µl DNase I (Worthington; 1,5 mM) and 60 µl collagenase/dispase (Roche; 20 mg ml^−1^) in ACSF. Vybrant dye (Thermo Fisher Scientific; Vybrant Ruby) and NeuO dye (STEMCELL Technologies) were added to the digestion mix. Vybrant Dye incorporates into nucleated cells to stain live neurons, whereas NeuO is a dye used for in vitro enrichment of neurons. We used the dye to further enrich for the neuronal cell population.

Ganglia were digested on a heating block at 37 °C with shaking for 1.5 h. Every 30 min, the cell suspension was further mechanically disrupted by pipetting up and down, starting with a 1-ml pipette and going down to a 200-ml pipette. As soon as all ganglia were dissociated, the cell suspensions were filtered using a 40-mm cell strainer (Falcon) and collected in a 15-ml plastic tube. The digestion solution was diluted with 10 ml RPMI medium containing 5% BSA and 1% fetal calf serum (FCS), and centrifuged at 100*g* for 4 min at 4 °C. The supernatant was removed, and the pellet was resuspended in 200 ml (CG) and 500 ml (DRG) RPMI medium containing 5% BSA and 1% FCS. The neurons innervating the tissue of interest were FACS-sorted, pre-gated on nucleated (Vybrant Ruby^+^) and NeuO^+^ cells, and were finally selected for FB signal. The cells were sorted in 384-well plates containing 1.2 ml lysis buffer (0.2% Triton, RNAse inhibitor, 10 µM polyTPrimer, 10 mM dNTPs). The plate was briefly centrifuged, snap-frozen and stored at −80 °C until further processing. For assessing AAV6 and FB single and co-labelling, cells were pre-gated on live cells and were analysed on the basis of FB and AAV6-mCherry signal.

### scRNA-seq of retrogradely labelled neurons using Smart-seq2.5

Single-cell libraries were generated according to the Smart-seq2.5 protocol^47^. In brief, cells were sorted in a 384-well plate containing 10 mM Triton X-100, oligo-dT primer (Smart-seq2 30 Oligo-dT Primer), dNTPs (NEB) and RNase inhibitor (Thermo Fisher Scientific). After annealing at 72 °C for 3 min on a thermal cycler, reverse transcription was performed in a master mix of Maxima RNaseH Minus RT enzyme and buffer (Thermo Fisher Scientific), PEG 7.5%, H_2_O, RNase inhibitor and a template switch oligonucleotide (SMART-seq2 50 TSO) using the following protocol: 42 °C for 90 min, followed by inactivation at 70 °C for 15 min. Whole-transcriptome amplification was achieved by adding KAPA HiFi HotStart ReadyMix (Kapa Biosystems) and IS PCR primer (ISPCR) to the reverse transcription product and amplification on a thermal cycler using the following protocol: 98 °C for 3 min, followed by 16 cycles of 98 °C for 20 s, 67 °C for 15 s and 72 °C for 6 min, followed by a final 5-minute extension at 72 °C. After ampure bead purification using the Bravo NGS System, the cDNA was assessed using a D5000 screen tape (Agilent), to confirm the expected size distribution and estimate the cDNA concentration. For the subsequent library preparation, cDNA was diluted to 0.33 ng µl^−1^. Tagmentation reactions were performed with the Nextera XT DNA Sample Preparation Kit (Illumina) using 100 pg of cDNA per single cell as input, with modified manufacturer’s instructions using the mosquito liquid handling system. In brief, 300 nl Nextera Xt, 600 nl TC buffer and 300 nl cDNA were transferred to a 384-well plate and incubated for 10 min at 55 °C. The reaction was stopped by adding 300 nl NT buffer. Libraries were indexed by PCR, pooled and purified twice with AMPure XP beads at a volume ratio of 0.9×, and the size distribution was assessed using a D1000 screen tape (Agilent TapeStation) and the Qubit High-Sensitivity DNA Kit (Invitrogen). Libraries were sequenced using NextSeq500/550 High Output v2 kits (75 cycles, Illumina) using single-end sequencing.

### scRNA-seq of retrogradely labelled neurons using an adapted version of Barcode-seq

Single-cell libraries were generated similarly to the SMART-seq2.5 protocol with small adjustments. In brief, RNA from single-cell lysates in barcoded oligo-dT primer, dNTPs (NEB) and RNase inhibitor (Thermo Fisher Scientific) were annealed at 72 °C for 3 min on a thermal cycler. Reverse transcription was performed in a master mix of Maxima RNAseH Minus RT enzyme and buffer (Thermo Fisher Scientific), PEG 7.5%, H_2_O, RNase inhibitor and a template switch oligonucleotide (SMART-seq2 TSO) using the following protocol: 42 °C for 90 min, followed by inactivation at 70 °C for 15 min. Whole-transcriptome amplification was achieved by adding KAPA HiFi HotStart ReadyMix (Kapa Biosystems) and IS PCR primer (ISPCR) to the reverse transcription product and amplification on a thermal cycler using the following protocol: 98 °C for 3 min, followed by 16 cycles of 98 °C for 20 s, 67 °C for 15 s and 72 °C for 6 min, followed by a final 5-minute extension at 72 °C. Samples from one 384-well plate were pooled, cDNA was purified with AMPure XP SPRI beads at a volume ratio of 0.7×, quality was assessed using a high-sensitivity DNA chip (Agilent Bioanalyzer), confirming the expected size distribution of 1,000–2,000 bp, and the sample was diluted to 1 ng μl^−1^. Tagmentation reactions were performed with the Nextera XT DNA Sample Preparation Kit (Illumina) using 1 ng of cDNA per 384 wells of pooled single cells as input, with modified manufacturer’s instructions as described. Libraries were purified with AMPure XP SPRI beads at a volume ratio of 0.7×, and the size distribution was assessed using a high-sensitivity DNA chip (Agilent Bioanalyzer) and Qubit High-Sensitivity DNA Kit (Invitrogen). Libraries were sequenced using NextSeq500/550 High Output v2 kits (75 cycles, Illumina) using paired-end sequencing. Barcode-seq enabled cost-effective sequencing of a large number of neurons, because individual cells are barcoded during annealing and can be pooled afterwards. However, because Smart-seq2 identified more genes per cell (Smart-seq, 10,124 genes per cell; Barcode-seq, 3,308 genes per cell) we used these data for most differential expression analysis, with Barcode-seq being an excellent tool for cell-type identification and further validation for analysis obtained with Smart-seq analysis (Extended Data Figs. 4 and 5).

### Cell lines

All cell lines used were authenticated by single-nucleotide polymorphism analysis and cells were regularly tested for mycoplasma contamination (Multiplexion).

### Human cells

Tumour patient-derived-cell lines (PDCs) have been previously reported^31^ or have been similarly derived (PACO10 and PACO43). PDCs were maintained in cancer stem cell (CSC) medium (TumorMACS, Miltenyi), grown in Primaria plates (Corning) and used at a maximum passage of 15.

Human primary pancreatic fibroblasts (Cell Biologics, H-6201) were cultured in fibroblast growth medium (PromoCell).

### Mouse cells

KPC cells were provided and produced as reported^48^. B16-F10 (ATCC) cells were bought and provided by collaboration partners. Cells were maintained in Dulbecco’s modified Eagle’s medium (DMEM; Thermo Fisher Scientific) with 5% FCS.

Mouse primary pancreatic CAFs were cultured in fibroblast growth medium (PromoCell).

### In vitro culture of mouse ganglia cells

Mouse CGs and DRGs were dissected as described above. The ganglion cells were washed in ice-cold ACSF, and the outer layer of the ganglion was removed under a stereomicroscope. For culturing ganglion cells, Primaria 24-well plates were used. A thin layer of Matrigel (Corning) (1:30 dilution in CSC medium) was plated on the well. One ganglion was further plated on the thin layer covered in 10 µl of 1:30 Matrigel dilution and incubated at 37 °C and 5% CO_2_. CSC (TumorMACS, Miltenyi) was added to the well and the cells were cultured in serum-free conditions at 37 °C and 5% CO_2_.

### In vitro co-cultures

Ganglia cells were seeded as described above and after axons started to grow out, cells were incubated for one hour with cell-trace violet (Thermo Fisher Scientific, C34557) to enable labelling. Cells were washed several times; fresh medium was added and PACO10-GFP (Sigma-Aldrich MISSION pLKO.1-puro-CMV-TurboGFP Positive Control Plasmid DNA) cells were added to the well. The cells were cultured together for three days before FACS-sorting of PACO cells. For cell proliferation assays, ganglion cells were seeded as described above and cancer cells (PACO10, PACO43 and KPC) and fibroblasts (human, CAFs) were cultured in a transwell chamber and seeded in 0.4-μm-pore transwells (Becton Dickinson). Cell numbers were adapted experimentally to ensure equal values during the experiment. The same number of cells was seeded in a transparent 24-well plate to monitor cell confluence during the experiment. Twenty-four hours later, fibroblasts were preincubated in CSC medium for one hour before setting the co-culture. Wells without ganglia served as controls. After three days (KPC), four days (PACO and human fibroblast) or five days (CAFs normalized on average duplication time) of co-culture, transwells were placed on a new plate and the number of cells was estimated using CellTiter-Blue stock solution (Promega) following the manufacturer’s instructions. Axonal sprouting could be measured using the Cytosmart imaging system. For conditioned medium experiments, CGs and DRGs were seeded as described above. After three days, the medium was changed, and cells were incubated in this medium for five days. PACO cells were seeded in a 24-well plate. Twenty-four hours later, the cell density was assessed using the Cytosmart readout, the medium was removed and new medium (1:1 fresh + supernatant of ganglia cells) was added. After three days of culture, the number of cells was estimated using CellTiter-Blue stock solution (Promega), following the manufacturer’s instructions and with Cytosmart quantification.

### IHC

Tumour or pancreas tissue was collected and washed in 10 ml PBS before fixation in 10 ml 4% PFA at 4 °C overnight. DRGs and CGs were subsequently dissected, washed in ACSF, fixed in 4% PFA for 20 min at room temperature, incubated overnight in sucrose and processed for cryosectioning.

Ten-millimetre serial cryosections were collected and processed for IHC. In brief, sections were washed with PBS and incubated with blocking buffer (PBS with 5% normal goat serum and 0.3% Triton X-100) for one hour at room temperature. The sections were then incubated with the primary antibody in the same blocking buffer overnight at 4 °C. The following day, sections were washed three times with wash buffer (PBS with 0.3% Triton X-100) before incubation with secondary conjugated antibody for one hour at room temperature. Sections were then washed three times with wash buffer before mounting in Fluoromount Aqueous Mounting Medium (Sigma). Samples were analysed under a Leica Zeiss confocal microscope.

### Xenografts

To generate orthotopic tumours of human PDX cells, 200,000 cells were mixed with Matrigel (2 mg ml^−1^; BD) and injected into the mouse pancreas. Engraftment of tumours and subsequent growth were monitored by regular palpation of the implantation site.

### Subcutaneous xenografts

To generate subcutaneous tumours of human PDX cells (or melanoma), 200,000 cells (or 50,000 cells) were mixed with Matrigel (2 mg ml^−1^; BD) and injected into the mouse flank or neck. Engraftment of tumours and subsequent growth were monitored by regular palpation of the implantation site.

### KPC allografts

To generate orthotopic tumours of mouse cells, 100,000 PDA30364 PDAC cells^48^ (mouse cell line derived from a *Elas-tTA/TetO-Cre;Kras*^*+/LSL-G12D*^;*Tp53*^+/LSL-R172H^ mouse line) were mixed with Matrigel (2 mg ml^−1^; BD) and injected into the mouse pancreas. Engraftment of tumours and subsequent growth were monitored by regular palpation of the implantation site.

### Intrapancreatic melanoma allografts

To generate intrapancreatic melanoma tumours of mouse cells, B16-F10 cells were mixed with Matrigel (2 mg ml^−1^; BD) and injected into the mouse pancreas. Engraftment of tumours and subsequent growth were monitored by regular palpation of the implantation site.

### Electroporation of the pancreas

To generate EPO KP tumours (EPO refers to an electroporation PDAC mouse model), we surgically exposed the pancreas through a lateral incision. Mice were anaesthetized using isoflurane 1.8% in air (v/v) and carprofen was injected. Bepanthen (Bayer) was applied to the eyes. Next, we injected a plasmid cocktail into the pancreas containing a transposon encoding *Kras*^*G12V*^, which integrates using an encoded transposase^49^, in combination with a plasmid that allows CRISPR-mediated *Tp53* inactivation, which models the most frequent driver mutations in PDAC. Ten microlitres of the plasmid cocktail in TE Endofree buffer (Qiagen Maxi- or Gigaprep kit) were injected into the pancreas using a 29G 20-µl Hamilton syringe. The bubble was grabbed and electroporated with a 5-mm² electrode. The orientation was switched and electroporated a second time (settings: voltage, 40 V; pulse interval, 500 ms; pulse length, 35 ms; number of pulses, 4)^49^. The efficiency of PDAC generation within three months was higher than 80%.

### Tissue digestion

Mouse pancreas, xenografts and electroporated tumours were digested using the Tumor Dissociation Kit (Miltenyi, 130-095-929) according to the manufacturer’s protocol.

In brief, tissue was collected and washed in ice-cold PBS. Pancreas was further blocked in RPMI + FCS and FCS was injected using a syringe to block digestion enzymes in the pancreas. The tissue was cut into small pieces and the digestion mixture was added to RPMI medium containing tumour or pancreas tissue. PDAC samples were digested according to the ‘tough tissue’ and pancreas according to the ‘soft tissue’ protocol. The samples were diluted with 40 ml RPMI medium, centrifuged and washed before further staining.

### FACS

Digested xenografts, electroporated tumour and mouse pancreas cells were stained with a total of three fluorescent cell surface antibodies and DAPI. Cells were sorted into four populations according to CD326^+^ (EPCAM^+^), EPCAM^−^CD31^+^, EPCAM^−^CD45^+^ and EPCAM^−^CD31^−^CD45^−^ expression using BD Aria or BD Fusion cell sorters. Cells from each population were sorted in PBS + 5% BSA and mixed in a 2:1:1:1 ratio. Thirty thousand cells of each sample were further processed for single-cell analysis.

Neurons, PACO10-GFP cells and human fibroblasts from co-culture experiments were sorted for GFP signal (PACO10), size and APC signal (neurons) from cell labelling using CellTracer dye. Fibroblasts were labelled before co-culture using CellTracer Blue (Thermo Fisher Scientific, C34574) and sorted for UV signal. Cells were sorted directly into RNA extraction buffer (Thermo Fisher Scientific, KIT0214), snap-frozen and stored at −80 °C until RNA extraction. Analytical flow-cytometry data were generated on BD Fortessa, BD LSRII and BD Symphony A5 with BD FACSDiva v.8.0.3 and were analysed using FlowJo v.10.5.3.

### RNA-seq of co-culture bulk samples

RNA extraction and purification of FACS-sorted cells was done using the PicoPure RNA Isolation Kit according to the manufacturer’s instructions (Thermo Fisher Scientific, KIT0214). RNA quality assessment and quantification were done with Bioanalyzer using the Agilent RNA 6000 Pico Kit (Agilent, 5067-1513). Whole-transcriptome amplification was performed using a modified Smart-seq2 protocol, with 5 μl of a modified RT buffer containing 1× SMART First Strand Buffer (Takara Bio Clontech, 639538), 1 mM dithiothreitol (Takara Bio Clontech), 1 μM template switching oligo (IDT), 10 U μl^−1^ SMARTScribe (Takara Bio Clontech, 639538) and 1 U μl^−1^ RNasin Plus RNase Inhibitor (Promega, N2615). Tagmentation of cDNA was done using the Nextera XT DNA Library Preparation Kit (Illumina, FC-121-1030). All RNA libraries were pooled and sequenced together on an Illumina NextSeq 550 high-output sequencer (1.4 pM with 1% PhiX loading concentration, single-end 75-bp read configuration).

### scRNA-seq using 10X Genomics

The single-cell analysis of full tumour and pancreas was performed with the 10X Genomics Chromium Single Cell Kit Version 3. Suspensions were prepared as described above and diluted in PBS; 30,000 cells were used as input and added to the 10X Chromium RT mix. For downstream cDNA synthesis (12–14 PCR cycles), library preparation and sequencing, we followed the manufacturer’s instructions.

### Sympathetic denervation with 6-OHDA

For sympathetic nerve disruption in mice, three doses of 6-OHDA (100 mg kg^−1^) or vehicle, were injected i.p. either three days before tumour induction (orthotopic and subcutaneous) or at the first time point at which a tumour was palpable.

### Ganglionectomy

NSG mice were anaesthetized using isoflurane. A carprofen depot (10 mg g^−1^) was injected subcutaneously 30 min before the surgery. Ganglionectomy was performed as described before^17^. In brief, a midline laparotomy was performed, and the pancreatic tail was exteriorized. After dissecting the pancreas until the level of the portal vein, the celiac artery and the superior mesenteric artery were identified. The CG was subtly resected using micro-surgical instruments under an operating microscope. The incision was checked for cessation of bleeding. Directly after, PDAC cells were injected into the pancreas as described above and the abdominal muscles was closed using 6-mm vicryl sutures (Ethicon).

### Botox denervation

For (sensory) nerve disruption in mice, NSG mice were anaesthetized after engraftment of PDX tumours using isoflurane. A carprofen depot (10 mg g^−1^) was injected subcutaneously 30 min before the surgery. Botulinum toxin (25 pg µl^−1^ in 5 μl PBS) was injected at five spots into the tumour mass to ensure sufficient distribution throughout the tumour and the abdominal muscles was closed using 6-mm vicryl sutures (Ethicon).

### In vivo drug treatment

NSG mice were palpated for any mass in the pancreas. Once there was a tumour palpable, the mice were randomly assigned to the control and treatment groups. Nab-paclitaxel (10 mg kg^−1^) was i.p. injected daily for five days followed by a nine-day drug holiday. This treatment scheme was performed twice for an early time point and four times for a late time point. Mice were injected with FB on the last treatment day and euthanized nine days later. Ganglia were processed as described above. Tumours were resected and measured by weight and size. For oxaliplatin treatment, Oxaliplatin (3 mg kg^−1^) was i.p. injected daily for five days followed by a nine-day drug holiday for two treatment rounds.

The ICI nivolumab (5 mg kg^−1^, twice weekly) was administered to BL6 mice by i.p. injection for 1 week after tumour establishment.

### In vivo drug and co-treatment

For sympathetic nerve disruption in mice, three doses of 6-OHDA or vehicle, 100 mg kg^−1^ per day were injected i.p. three days before tumour induction. NSG mice were palpated for any mass in the pancreas or for a subcutaneous tumour, respectively. Once there was a tumour palpable, nab-paclitaxel (10 mg kg^−1^) was i.p. injected as described above for three rounds (with five days of treatment and nine treatment holidays) or two rounds (with four days of treatment and ten treatment holidays), respectively. Mice were euthanized nine days after the last treatment. Tumours were resected and measured by weight and size. Control mice received either only nab-paclitaxel or the vehicle control. For co-treatment with botox, mice were anaesthetized, and botox was administered into the tumour by injection as described above. After closing of the wound, nab-paclitaxel was administered for two rounds with four days of treatment and ten treatment holidays.

For co-treatment with oxaliplatin, oxaliplatin was administered i.p. for two treatment rounds as described above after 6-OHDA denervation.

For co-treatment in BL6 mice, 6-OHDA was used as described above and nivolumab (5 mg kg^−1^, twice weekly) was i.p. injected for 1 week.

### Primary and secondary tumour growth after resection

To assess primary and secondary tumour establishment and the neuronal effects, mice were anaesthetized and operated on as described above. Mice were either injected with PACO10 cells (in Matrigel) by injection in the pancreas tail or sham-injected. After tumour establishment, mice were anaesthetized and tumour mass or sham-operated pancreas was exposed. Two millimetres before the tumour, or at the same location on the pancreas in the sham-operated control mice, the pancreas was closed by stitches, and tumours, or part of the healthy pancreas in control mice, were resected. A secondary, or, in the case of the pancreatomy, primary, tumour was established by injecting PACO10 cells into the remaining pancreas tissue (head). Engraftment of tumours and subsequent growth were monitored by regular palpation of the implantation site. At the end-point, mice were euthanized, and tumours were extracted.

### Resection followed by Trace-n-Seq

To assess preservation of PCN signature after tumour resection, mice were anaesthetized and operated on as described above. Mice were either injected with PACO10 cells (in Matrigel) by injection in the pancreas tail or sham-injected. After tumour establishment, mice were anaesthetized and tumour mass or sham-operated pancreas was exposed. FB was administered into the tumour or into the same location in the sham-operated pancreas as described above. Seven days later, after sufficient tracing of tumour- or sham-pancreas-innervating neurons, 2 mm before the tumour, or at the same location on the pancreas in the sham-operated control mice, the pancreas was closed by stitches, and tumours, or part of the healthy pancreas in control mice, were resected. The wound was closed and 28 days later mice were euthanized and subjected to Trace-n-Seq-based sorting of FB^+^ neurons (now 28 days absent of tumour innervation). Mice were analysed for any remaining tumour to assess sufficiency of the resection. All mice were tumour-free at the time point of the euthanasia.

### Model of acute pancreatitis

Pancreas-innervating neurons in mice (BL6) were traced using FB as described above. Seven days later, mice were injected hourly with 100 μl of 50 μg kg^−1^ cerulin in 0.9% NaCl for 10 h to introduce acute pancreatitis. Control mice received only NaCl injections. Sixteen hours after the last injection, mice were euthanized and ganglia were analysed by single-cell sequencing as described above.

### Staining of human specimens

For the cohort that was neoadjuvantly treated with nab-paclitaxel (*n* = 10 cases), a matching cohort was generated for gender, age group (within a 10-year range, ±5 years) and primary tumour location, including chemotherapy-naive PDAC, FOLFIRINOX-neoadjuvant-treated PDAC and healthy pancreas, using formalin-fixed paraffin-embedded (FFPE) tissue from the archives of the Pathology Institute in Heidelberg. For the healthy pancreas cases, tissue was selected from patients with a non-malignant diagnosis (for example, serous cystic neoplasia), away from the lesion and without any sign of chronic pancreatitis or in situ neoplasia. The chemotherapy-naive and FOLFIRINOX-treated PDAC tissue selection included representative intrapancreatic and peripancreatic tumour compartments. Neoadjuvant-treated PDAC tissue was selected on the basis of the most representative predominantly intrapancreatic FFPE block of the case, independently of the regression evidence. Selected FFPE blocks were 3-mm sectioned and stained using the standardized assay for neurofilament (clone 2F11 mouse monoclonal, Cell Marque with a concentration 0,09 µg ml−^1^) using a Ventana Benchmark Ultra platform (Ventana Medical Systems). Langerhans islets normally present were used as an internal control.

One whole section of each case was annotated for one as-large-as-possible region of interest (ROI), which was subclassified as ‘pancreas tissue’; ‘PDAC’ and ‘NAC-PDAC_1(NAB-PACLITAXEL)’. All morphologically identifiable nerve fascicles, independent of size and neurofilament (NF) intensity of staining, as well as all NF-positive nerve fibres, were annotated using Q-Path (v.0.3.2, downloaded 15 September 2022).

For human validation of PCN-signature genes, 3-mm whole-tissue sections of the cohort were stained for ROBO2 (Novus Biologicals, clone NBP1-81399, rabbit polyclonal, 1:100), TH (Invitrogen, clone MA1-24654, mouse monoclonal, 1:50) and CGRP (Abcam, clone ab81887, mouse monoclonal, 1:100) using the Ventana Benchmark Ultra platform (Ventana Medical Systems). One ROI corresponding to the highest quality area among all stains was manually drawn. All morphologically identifiable nerve fascicles, independent of size and staining intensity, including unstained nerve fibres, were annotated using Q-Path (v.0.3.2, downloaded 15 September 2022). Nerve structures in the ROBO2-stained sections were annotated using a two-tiered categorization to further separate ROBO2-negative from ROBO2-positive nerves. Cell detection and cell classifications were performed using QuPath v.0.5.1. For NF and TH, manual annotations were used to train cell classifiers with QuPath, whereas for ROBO2 and CGRP, manual annotations were used. Downstream data analysis was performed in R v.4.0.3.

### Quantification using PCP

Image analysis software: commercial software (Aivia 10.5.1, Leica Microsystems) was used to apply the random-forest machine-learning algorithm. By painting ROIs of the different neural markers and background in a single image, the pixel classifier was trained. The set used for training includes the most common image transforms at medium and large kernel sizes (Gaussian, Hessian, Laplacian and structural tensor). Using an interactive process and fast preview of results allowed us to create a model that is then applied to the rest of the images. Thresholding was adapted for each resulting confidence map.

For quantifying the tumour areas (or tumour masses, areas or tissue) the light-sheet-microscopy volumes were projected in the plane using the maximum intensity projection method in Fiji v.2.0. Following the same steps as for the neural markers, another model was created by training the classifier with annotations for tumour mass and background. The values obtained for the different neural markers were then normalized to the tumour mass of each sample. Videos and animations were created using the commercial software Aivia.

### Alignment of scRNA-seq data and gene-expression quantifications

For Smart-seq2 data, raw sequencing reads were aligned to the reference genome (mm10, GRCm38) using STAR (v.2.5.3a)^50^ and gene expression was quantified using htseq-count (v.2.0.1)^51^. For the Barcode-seq data, data were aligned to the mm10 genome, quantified and split into cell barcodes using STAR and the BRB-Seq pipeline (v.3.1, https://github.com/DeplanckeLab/BRB-seqTools)^52^. For bulk RNA-seq data, reads were aligned to a combined mouse and human genome using STAR (mm10 and hg38, GRCh38) and quantified using htseq-count, equivalently to the Smart-seq2 data. For 10X scRNA-seq data, Cell Ranger (v3.1.0) was used against a combined mm10 and hg38 index with default options. Analysis was performed in RStudio v.3.5.2 (https://www.r-project.org).

### Low-level analysis of scRNA-seq data

Low-level analysis of scRNA-seq data was performed mostly using functions from the scran (v.1.20.1) and scater (v.1.20.1) R packages^53^. First, cells with fewer than 50,000 mapped reads and 4,000 detected genes and cells with more than 20% of reads mapping to mitochondria were removed. Next, counts were normalized using the computeSumFactors function and log-transformed. The resulting gene-expression matrix was used for dimensionality reduction by principal component analysis (prcomp, stats), *t*-distributed stochastic neighbour embedding (*t*-SNE) (Rtsne, Rtsne, v.0.15) and uniform manifold approximation and projection (UMAP) (umap, umap, v.0.2.7.0). To identify clusters, we used graph-based community detection using the Louvain algorithm implemented by the functions buildSNNGraph and cluster_louvain of the package igraph (v.1.2.10).

### Cell-type annotation of single neurons

Cell types were annotated using a label-transfer approach from a previously annotated reference dataset^26,27^. To this end, we identified highly variable genes using the findTopHVGs function from the scran package in the query dataset, and used only these genes for further annotation. We then computed pairwise Pearson correlation coefficients between each query and each reference cell type and annotated each query cell as the cell type with the highest correlation coefficient. We also annotated the dataset using the SingleR function in the SingleR package (v.1.6.1)^54^ and found high concordance between the two approaches. We took both main and sub cell types, as well as neurotransmitter status, from a previous dataset^26^, and furthermore annotated the DRG neurons with a separate reference dataset^27^ with higher resolution. To visualize the integration, we used an MNN-based correction algorithm^55^ to integrate the reference and query datasets, subset to the intersection of the 1,000 most highly variable genes each and computed a joint UMAP.

### Differential expression analysis

To identify DEGs between pancreas- and tumour-innervating neurons, we used DESeq2 (v.1.32.0) on pseudobulk libraries. Pseudobulks were generated across experimental replicates (*n* = 2 for healthy and *n* = 3 for cancer). Genes were considered significantly differentially expressed when they showed *P*_adj_ < 0.1. DEG analysis was performed separately for whole-ganglion aggregates and individual cell types. For some analyses, DEGs are considered as genes with *P*_adj_ < 0.1 in a Wilcoxon rank sum test when comparing individual cells, owing to the lack of biological replicates.

DEG analysis was performed in an analogous manner for the bulk RNA-seq experiment of co-cultured PDAC cells, fibroblasts and ganglia. GSEA was performed using GSEA_4.0.3 (Broad Institute).

### Generation of the PCN signature

To generate a signature to identify expression changes in pancreatic-cancer-infiltrating neurons, the top 500 up- and downregulated genes for all five major subopulations on the basis of the DEG analysis presented in Fig. 2 were selected. To include only robustly expressed genes, genes with a mean expression value of less than 500 or *P*_adj_ > 0.2 in a population were excluded. For the PCN-down signature, all genes identified in three or more populations, and for the PCN-up signature, genes identified in two or more populations, were included in the signature, and genes detected only in CG populations were excluded.

### Analysis of 10X scRNA-seq of sorted pancreatic and PDAC stromal cells

10X scRNA-seq data were processed by first removing cells with fewer than 1,000 unique molecular identifiers and fewer than 500 detected genes, as well as those with higher than 10% mitochondrial RNA content. For the tumour-bearing sample, we identified cancer cells as cells with mainly reads aligning to the human genome. We used computeSumFactors for size-factor normalization and Louvain clustering for community detection, similarly to the Smart-seq2 data analysis. We then assigned cell types on the basis of marker genes. Finally, we visualized the datasets using dimensionality reduction by UMAP on the first 50 principal components. We processed the healthy pancreas and the tumour-bearing sample separately.

### Interaction potential analysis

The interaction potential analysis quantifies the total expression of receptors and ligands in potential target cells, for which the cognate receptor or ligand is expressed in innervating neurons. We closely followed the approach outlined previously^43^. We first identified expressed receptors or ligands in neuronal subpopulations by obtaining gene lists encoding receptors and ligands from the SingleCellSignalR (v.1.4.0)^56^ and considered them expressed in neurons if their log aggregate read counts exceeded 2. We then calculated the *z*-scored mean expression of all cognate genes in interaction partners and subtracted the minimum interaction score to yield a final interaction potential. We performed the analysis by comparing pancreas-innervating neurons with stroma from healthy mice and tumour-innervating neurons with PDAC cells and associated stroma, and individually per biological replicate. We then calculated the product of neuronal and *z*-scored interaction-partner gene expression for healthy-pancreas-innervating neurons and pancreatic stroma, and for cancer-innervating neurons and PDAC stroma, to identify individual gene pairs interacting with the largest differences in these interaction scores.

### Statistics and reproducibility

Unless otherwise specified, cryosection staining of ganglia was performed with *n* = 3 mice, with a minimum of three slides imaged per ganglion (for marker validation, tracer analysis and subtype identification). AAV tracing experiments were performed using one mouse per time point and AAV type (for microscopy and FACS), with each condition repeated twice. For co-injection experiments (double pancreas and pancreas plus spleen), two mice were used in independent experiments. scRNA-seq using 10X Genomics was performed on three pancreatic samples (processed in two experiments; three libraries), four PDAC control samples (processed in two experiments; four libraries) and three denervated PDAC samples (processed in one experiment; three libraries). Tumour size experiments were performed with at least five mice per condition. Key experiments, such as combination treatment with paclitaxel and 6-OHDA (two repetitions with varying dosages) and 6-OHDA denervation (six repetitions), were repeated individually. Tracing experiments (flow cytometry) were performed in a minimum of three mice per condition. Data comparisons between healthy and tumour samples were done for every experiment to ensure data robustness. FACS analysis of stromal compartments after denervation was repeated three times, with three to five mice per experiment. LSFM staining frequencies are detailed elsewhere in the Methods. For all staining experiments that were quantified, data and sample sizes are indicated in the respective figure legends of the quantification plot. For IHC, the number of patient samples used is specified in the quantification plots. Neurofilament staining was independently reproduced twice. For each human experiment, all markers were stained twice for each patient sample. Statistical analysis was performed using Prism versions 9–10.3.1. For figure generation, we used Adobe Illustrator 2024 (v.28.7.1. Adobe) and BioRender (www.biorender.com).

### Reporting summary

Further information on research design is available in the Nature Portfolio Reporting Summary linked to this article.

## Online content

Any methods, additional references, Nature Portfolio reporting summaries, source data, extended data, supplementary information, acknowledgements, peer review information; details of author contributions and competing interests; and statements of data and code availability are available at 10.1038/s41586-025-08735-3.

## Supplementary information

Supplementary Discussion This file contains Supplementary Discussion, including additional references.

Reporting Summary

Supplementary Tables Supplementary Tables 1–21.

Supplementary Video 1 Innervation from the celiac plexus to pancreas and spleen via LSFM. The pancreas with attached spleen including the celiac plexus was cleared with iDISCO and stained using peripherin antibody. The axons projecting from the celiac plexus to/and through pancreas into spleen is shown.

Supplementary Video 2 DRG in the spine via LSFM. The spine was cleared with iDISCO and stained using peripherin antibody. Individual DRG and nerve bundles are shown.

Supplementary Video 3 Innervation in the healthy pancreas via LSFM. The pancreas was cleared with iDISCO and stained using peripherin antibody. The axons projecting through the pancreas are shown.

Supplementary Video 4 Innervation in PDAC via LSFM. PDX PDAC was cleared with iDISCO and stained using peripherin antibody. The axons projecting through the tumour are shown.

## Source data

Source Data Figs. 1–5 and Source Data Extended Data Figs. 1, 2, 6–8, 11, 13–15.

## Data Availability

All newly generated sequencing datasets have been deposited in ArrayExpress under the following accession numbers: scRNA-seq of traced neurons using Smart-seq2 (E-MTAB-12940), scRNA-seq of traced neurons using Barcode-seq (E-MTAB-12941), bulk RNA-seq of co-cultured fibroblasts and cancer cells with ganglia (E-MTAB-12899) and Chromium 10X scRNA-seq of stromal cells in pancreas and xenografts (E-MTAB-12906). Source data are provided with this paper.
