# Supplementary material for: Characterization of single neurons reprogrammed by pancreatic cancer
Source: Nature. 2025 Feb 17;640(8060):1042–51. doi: 10.1038/s41586-025-08735-3 (PMC12018453; doi:10.1038/s41586-025-08735-3)
Supplement: Supplementary file 1 — This file contains Supplementary Discussion, including additional references. [file 41586_2025_8735_MOESM1_ESM.pdf]

nature portfolio

https://doi.org/10.1038/s41586-025-08735-3

Supplementary information

Characterization of single neurons reprogrammed by pancreatic cancer

In the format provided by the authors and unedited

Supplementary discussion

Our study addresses molecular changes in PDAC and allows characterization of organ-specific neurons in health and disease with single cell resolution. Other dyes or AAVs are limited by tropism, duration of viral transport or labor-intensive microscopy-based cell picking[1-4]. Trace-n-Seq, enables high-throughput profiling of thousands of neurons for downstream processing with various pipelines, deconvoluting extensive neuronal heterogeneity[5,6]. First, within our pancreas and PDAC neuron reference, we detect transcriptional programs relate to injury, neural regeneration, and development unique in their sum. The Pancreatic Cancer-Nerve (PCN) signature derived from this persists even after tumor resection or exposure to neurotoxic drugs. This nerve-state can boost tumor proliferation and may facilitate survival and proliferation of residual tumor cells driving local relapse in patients. Second, neurons alter their surrounding TME. They induce proliferation of tumor cells and CAFs with immunosuppressive phenotypes. Experimental denervation induced a pro-inflammatory TME, extending studies that suggest denervation in combination with ICI treatments and neuron-fibroblast interactions. As neurons express PDL1, direct and indirect effects need addressing[7,8]. The proposed molecular interactions allow future functional studies to dissect Semaphorins, Ephrins and other factors roles in how “cancer cell-neuronal-TME” networks promote.

Third, in PDAC, although some nociceptors were present, most sensory neurons were of the NEFM subtype, different to the healthy pancreas. This may explain why very early-stage PDAC is usually painless, despite its hyperinnervation, and therefore often remains undetected until reaching advanced stages. This shines a new light on visceroceptive proprioceptors and light touch/vibration sensing neurons also described in intestine and other organ innervating neurons[1,9-11]. Their function remains elusive, but they may sense or influence tissue stiffness, raising potential roles for neuronal mechanosensation in cancer. The cancer-neuron footprint may affect organs beyond the tumor, as neurons spread e.g. to the spleen, potentially modulating neuro-immune or neuro-endocrine interactions[1,7,12]. Similar integration of tumors into neuronal networks exists in the CNS[13-17].

Fourth, the cancer-nerve state is a potential drug target. It is maintained after resection, suggesting peripheral neuron memory, warranting investigation into its epigenetic, electrophysiological and functional properties. Oncologists have unknowingly targeted cancer-nerves for decades, although ineffectively, as nab-paclitaxel, but not oxaliplatin, reduces PDAC innervation, particularly of sensory neurons. Synergistics effects of targeting sympathetic (6-OHD) and sensory (nab-paclitaxel) neurons underscores a role of both for PDAC growth. This supports observations that taxane neuropathy correlates with superior outcomes in PDAC[18]. Hence, pharmacological/surgical blockade of nerve-tumor cell communication, with chemotherapy and/or immune therapy, provides rapidly translatable opportunities to target PDAC.

More broadly, visualization, molecular characterization, and functional dissection of how cancers exploit the PNS remains a key research goal. Trace-n-Seq enables single cell assessment of this intricate relationship. These advancements will lead to novel treatments disrupting the cancer-nerve axis.

1 Hockley, J. R. *et al.* Single-cell RNAseq reveals seven classes of colonic sensory neuron. *Gut* **68**, 633-644 (2019).
2 Huang, S. *et al.* Lymph nodes are innervated by a unique population of sensory neurons with immunomodulatory potential. *Cell* **184**, 441-459. e425 (2021).
3 Fontaine, A. K. *et al.* Optical vagus nerve modulation of heart and respiration via heart-injected retrograde AAV. *Scientific reports* **11**, 1-12 (2021).
4 Veerakumar, A., Yung, A. R., Liu, Y. & Krasnow, M. A. Molecularly defined circuits for cardiovascular and cardiopulmonary control. *Nature*, 1-8 (2022).
5 Zeisel, A. *et al.* Molecular architecture of the mouse nervous system. *Cell* **174**, 999-1014. e1022 (2018).
6 Sharma, N. *et al.* The emergence of transcriptional identity in somatosensory neurons. *Nature* **577**, 392-398 (2020).
7 Balood, M. *et al.* Nociceptor neurons affect cancer immunosurveillance. *Nature*, 1-8 (2022).
8 Globig, A. M. *et al.* The beta(1)-adrenergic receptor links sympathetic nerves to T cell exhaustion. *Nature* **622**, 383-392 (2023). https://doi.org/10.1038/s41586-023-06568-6
9 Servin-Vences, M. R. *et al.* PIEZO2 in somatosensory neurons controls gastrointestinal transit. *Cell* **186**, 3386-3399 e3315 (2023). https://doi.org/10.1016/j.cell.2023.07.006
10 Wolfson, R. L. *et al.* DRG afferents that mediate physiologic and pathologic mechanosensation from the distal colon. *Cell* **186**, 3368-3385 e3318 (2023). https://doi.org/10.1016/j.cell.2023.07.007
11 Marshall, K. L. *et al.* PIEZO2 in sensory neurons and urothelial cells coordinates urination. *Nature* **588**, 290-295 (2020). https://doi.org/10.1038/s41586-020-2830-7
12 Zhao, Q. *et al.* A multidimensional coding architecture of the vagal interoceptive system. *Nature* **603**, 878-884 (2022).
13 Hausmann, D. *et al.* Autonomous rhythmic activity in glioma networks drives brain tumour growth. *Nature*, 1-8 (2022).
14 Pan, Y. *et al.* NF1 mutation drives neuronal activity-dependent initiation of optic glioma. *Nature* **594**, 277-282 (2021).
15 Venkataramani, V. *et al.* Glutamatergic synaptic input to glioma cells drives brain tumour progression. *Nature* **573**, 532-538 (2019).
16 Venkatesh, H. S. *et al.* Electrical and synaptic integration of glioma into neural circuits. *Nature* **573**, 539-545 (2019).
17 Venkatesh, H. S. *et al.* Targeting neuronal activity-regulated neuroligin-3 dependency in high-grade glioma. *Nature* **549**, 533-537 (2017).
18 Goldstein, D. *et al.* Development of peripheral neuropathy and its association with survival during treatment with nab-paclitaxel plus gemcitabine for patients with metastatic adenocarcinoma of the pancreas: A subset analysis from a randomised phase III trial (MPACT). *European Journal of Cancer* **52**, 85-91 (2016).
