# Supplementary material for: Characterization of single neurons reprogrammed by pancreatic cancer
Source: Nature. 2025 Feb 17;640(8060):1042–51. doi: 10.1038/s41586-025-08735-3 (PMC12018453; doi:10.1038/s41586-025-08735-3)
Supplement: Supplementary file 2 — Reporting Summary [file 41586_2025_8735_MOESM2_ESM.pdf]

Corresponding author(s): Andreas Trumpp
Last updated by author(s): 2024/11/02

Reporting Summary

Nature Portfolio wishes to improve the reproducibility of the work that we publish. This form provides structure for consistency and transparency in reporting. For further information on Nature Portfolio policies, see our Editorial Policies and the Editorial Policy Checklist.

Statistics

For all statistical analyses, confirm that the following items are present in the figure legend, table legend, main text, or Methods section.

n/a	Confirmed	
☐	☒	The exact sample size (*n*) for each experimental group/condition, given as a discrete number and unit of measurement
☐	☒	A statement on whether measurements were taken from distinct samples or whether the same sample was measured repeatedly
☐	☒	The statistical test(s) used AND whether they are one- or two-sided<br>*Only common tests should be described solely by name; describe more complex techniques in the Methods section.*
☐	☒	A description of all covariates tested
☐	☒	A description of any assumptions or corrections, such as tests of normality and adjustment for multiple comparisons
☐	☒	A full description of the statistical parameters including central tendency (e.g. means) or other basic estimates (e.g. regression coefficient) AND variation (e.g. standard deviation) or associated estimates of uncertainty (e.g. confidence intervals)
☐	☒	For null hypothesis testing, the test statistic (e.g. F, t, r) with confidence intervals, effect sizes, degrees of freedom and P value noted<br>*Give P values as exact values whenever suitable.*
☒	☐	For Bayesian analysis, information on the choice of priors and Markov chain Monte Carlo settings
☒	☐	For hierarchical and complex designs, identification of the appropriate level for tests and full reporting of outcomes
☐	☒	Estimates of effect sizes (e.g. Cohen's *d*, Pearson's *r*), indicating how they were calculated

Our web collection on statistics for biologists contains articles on many of the points above.

Software and code

Policy information about availability of computer code

Data collection	BD FACSDiva v8.0.3 (Flowcytometers and sorters, BD Bioscience), ZEN blue v2.5 (Zeiss international)
Data analysis	FlowJo (Versions 10.5.3), statistical analysis with Graphpad Prism (versions 9), R-studio v3.5.2 (www.r-project.org), for all packages used see methods, GSEA software v4.0.3 (Broad institute), FIJI v.2.0, Aivia 10.5.1 (Leica microsystems, Bellevue, WA), Q-Path (version 0.3.2, downloaded at 15.09.2022), TeraStitcher within ImSpector (Version 7.3.1) (Lavision GmbH), NIS-Elements (version 5.21, 5.41 and 5.42 Nikon GmbH, Germany)<br><br>For figure generation, we used Adobe Illustrator 2024 (version 28.7.1. Adobe) and biorender (www.biorender.com). Figure licences are provided in the Supplementary table

For manuscripts utilizing custom algorithms or software that are central to the research but not yet described in published literature, software must be made available to editors and reviewers. We strongly encourage code deposition in a community repository (e.g. GitHub). See the Nature Portfolio guidelines for submitting code & software for further information.

Data

Policy information about availability of data

All manuscripts must include a data availability statement. This statement should provide the following information, where applicable:

- Accession codes, unique identifiers, or web links for publicly available datasets
- A description of any restrictions on data availability
- For clinical datasets or third party data, please ensure that the statement adheres to our policy

Data Availability
All newly generated sequencing datasets have been deposited in ArrayExpress under the following accession numbers: Single-cell RNA-Sequencing of traced neurons using Smart-Seq2 (E-MTAB-12940), single-cell RNA-Sequencing of traced neurons using Barcode-Seq (E-MTAB-12941), bulk RNA-Seq of co-cultured fibroblasts and cancer cells with ganglia (E-MTAB-12899) and Chromium 10x single-cell RNA-Sequencing of stromal cells in pancreas and xenografts (E-MTAB-12906). Nucleotide sequences and all other source data is available upon reasonable request from the corresponding author.

External datasets used:
mm10 (Mouse genome), Link:https://www.ncbi.nlm.nih.gov/datasets/genome/GCF_000001635.20/

Published RNA sequencing datasets investigated:
Surgical Denervation 1:Renthal et al. Neuron 2020
Surgical Denervation 2:Wang et al. Cell research 2021
Surgical Denervation 3: Chandran et al. Neuron 2016
Inflammatory Stress: Liu et al., Frontiers in Cellular Neuroscience 2022
Sympathetic trunc data: Furlan et al. Nature neuroscience 2016
Reference Dataset for annotations 1: Zeisel et al. Cell 2018
Reference Dataset for annotations 2: Sharma et al.Nature 2020

Research involving human participants, their data, or biological material

Policy information about studies with human participants or human data. See also policy information about sex, gender (identity/presentation), and sexual orientation and race, ethnicity and racism.

Reporting on sex and gender	Sex/Gender for human samples was not a focus of investigation, but is included in Supplemental Table 12. Mouse studies were repeated with male mice to ensure similar findings betweens sexes.
Reporting on race, ethnicity, or other socially relevant groupings	does not apply
Population characteristics	Population characteristics, Age, Gender/Sex/Tumor stage(TNM) /neoadiuvant treatment cycles and tumor location are reported in supplemental Table 12.
Recruitment	does not apply
Ethics oversight	Human tissue samples were obtained and approved by the ethical committee of the University of Heidelberg (case number S-206/2011, S-206/2011, 206/2005) and conducted in accordance with the Helsinki Declaration; written informed consent was obtained from all patients.

Note that full information on the approval of the study protocol must also be provided in the manuscript.

Field-specific reporting

Please select the one below that is the best fit for your research. If you are not sure, read the appropriate sections before making your selection.

☒ Life sciences ☐ Behavioural & social sciences ☐ Ecological, evolutionary & environmental sciences

For a reference copy of the document with all sections, see nature.com/documents/nr-reporting-summary-flat.pdf

Life sciences study design

All studies must disclose on these points even when the disclosure is negative.

Sample size	Sample size was determined based on extensive experience with similar experiments in our laboratory (Noll et al., nature medicine 2016).
Data exclusions	No data was exluded.
Replication	All replicates have been included in the manuscript. Effect were always reproduced.

Replication

Biological replicates / Independent experiments were applicable are listed within the figure legends/Figures.
We additionally provide a data reproducibility statement in the methods section.
"Unless otherwise specified, cryosection staining of ganglia was performed with an n=3 mice, with a minimum of 3 slides imaged per ganglion (for marker validation, tracer analysis, and subtype identification). AAV tracing experiments were conducted using one mouse per timepoint and AAV type (for microscopy and FACS), with each condition repeated twice. For co-injection experiments (double pancreas and pancreas +spleen), 2 mice were used in independent experiments. Single-cell RNA sequencing using 10x Genomics was performed on 3 pancreatic samples (processed in 2 experiments, 3 libraries), 4 PDAC control samples (processed in 2 experiments, 4 libraries), and 3 denervated PDAC samples (processed in 1 experiment, 3 libraries). Tumor size experiments were conducted with at least 5 mice per condition. Key experiments, such as combination treatment with paclitaxel/6OHDA (2 repetitions with varying dosages) and 6OHDA denervation (6 repetitions), were repeated individually. Tracing experiments (flow cytometry) were performed in a minimum of 3 mice per condition. Data comparison between healthy and tumor samples was collected from every experiment to ensure data robustness. FACS analysis of stromal compartments post-denervation was repeated 3 times, with 3-5 mice per experiment. LSFM staining frequencies are detailed in the methods section. For all staining experiments that were quantified data and sample sizes are indicated in the respective figure legends of the quantification plot. For IHC, the number of patient samples used is specified in the quantification plots. Neurofilament staining was independently reproduced twice. For each human experiment, all markers were stained twice for each patient sample."

Randomization

All samples/ mice were analysed and allocated randomly.

Blinding

No blinding occured, as the experiments were performed by the same researchers, requiring sample annotation by the same persons making annonymous blinding imposible.

Reporting for specific materials, systems and methods

We require information from authors about some types of materials, experimental systems and methods used in many studies. Here, indicate whether each material, system or method listed is relevant to your study. If you are not sure if a list item applies to your research, read the appropriate section before selecting a response.

Materials & experimental systems

n/a	Involved in the study	
☐	☒	Antibodies
☐	☒	Eukaryotic cell lines
☒	☐	Palaeontology and archaeology
☐	☒	Animals and other organisms
☐	☒	Clinical data
☒	☐	Dual use research of concern
☒	☐	Plants

Methods

n/a	Involved in the study	
☒	☐	ChIP-seq
☐	☒	Flow cytometry
☒	☐	MRI-based neuroimaging

Antibodies

Antibodies used

Primary antibodies

Antigene Source Catalogue number Clonality Clone Validated target species Validation tissues Antibody dilution

Peripherin Merck AB1530 Polyclonal - mouse Celiac Ganglion 1:100
Peripherin abcam ab39374 Monoclonal SP1 mouse Celiac Ganglion 1:100
TH Santa Cruz sc-25269 Monoclonal F-11 mouse Celiac Ganglion 1:100
Tyrosine Hydroxylase abcam ab75875 recombinant EP1533Y human Celiac Ganglion 1:100
SHOX2 St John's Laboratory Ltd STJ194828 Polyclonal - mouse Adrenal gland 1:100
CD326 (EpCAM)-FITC Miltenyi 130-113-263 Monoclonal HEA-125 human human cancer cell lines 1:100
LIN28B LSBio LS[illegible]C748024 Polyclonal - mouse Fetal mouse brain 1:100
SLIT2 ThermoFisher PA5-31133 Polyclonal - mouse Fetal mouse brain 1:100
beta III Tubulin abcam ab18207 Polyclonal - mouse Celiac Ganglion 1:100
CGRP abcam ab36001 Polyclonal - mouse Adrenal gland 1:100
Socs 2 ThermoFisher BS-1896R Polyclonal - mouse Fetal mouse brain 1:100
SEMA5A ThermoFisher PA5-47791 Polyclonal - mouse Fetal mouse brain 1:100
APC CD45 BioLegend 103111 Monoclonal 30-F11 mouse Blood 1:100
ROBO2 Novus biologicals NBP1-81399 Polyclonal - human Fetal mouse brain 1:100
Thyrosin hydroxylase Invitrogen MA1-24654 Monoclonal 185 mouse human Celiac Ganglion 1:100
CGRP Abcam ab81887 Monoclonal 4901 mouse human Dorsal root ganglion 1:100
Neurofilament ThermoFisher Z2091MS Monoclonal 2F11 human Adrenal gland 1:100

Secondary antibodies
Donkey Anti-Goat IgG H&L abcam ab150129 1:500
Goat Anti-Chicken IgY H&L (Alexa Fluor 647) abcam ab150171 1:500
Alexa Fluor® 647 AffiniPure Donkey Anti-Chicken IgY (IgG) (H+L) Jackson Immuno Research/Biozol 703-605-155 1:500

Validation

Antibodies were used based on known cell type specific expression patterns, previous expertice and research in the laboratory and

Validation	the manufacturares instructions. Tissues used for validation of primary antibodies are listed in the table as well as in supplemental Table 1

Eukaryotic cell lines

Policy information about cell lines and Sex and Gender in Research

Cell line source(s)	Cell lines were previously established in our laboratory from primary human PDAC samples (Noll et al., Nature Medicine 2016). KPC cells were generated from tumor bearing mice (Bauman et al., Nature Communications 2022), B16F10 cells were provided by the group of Prof. Helmut Augustin and previously pruchased from ATCC.
Authentication	Primary cells lines were established in our laboratory and screened for known markers before use in experiments.
Mycoplasma contamination	cell lines did not contain mycoplasms
Commonly misidentified lines (See ICLAC register)	No commonly missidentified cell lines were used for this study.

Animals and other research organisms

Policy information about studies involving animals; ARRIVE guidelines recommended for reporting animal research, and Sex and Gender in Research

Laboratory animals	Wild-type mice had a C57BL/6 (BL6) background (Janvier Labs). NOD.Prkdcscid.Il2rgnull (NSG) mice were bred and housed under specific pathogen-free conditions in IVC cages at the central animal facility of the German Cancer Research Center (DKFZ) Humidity: 50%, Temperatur: 22°C, with normal Dark/light cycles.<br>Mouse age at experiment start was 8-24 weeks.
Wild animals	No wild animals were used in this study
Reporting on sex	only female animals were used in this study to increase reproducibility, for one specific experiment tumor were generated in male mice to compare changes between male and female animals
Field-collected samples	No wild field-collected samples were used in this study
Ethics oversight	All animal procedures were conducted in accordance with the guidelines of Germany and approved by the Ethics Committee for animal experimentation of Karlsruhe (Regierungspräsidium Karlsruhe; G230/19, G49/19, G105/17, and G148/21).

Note that full information on the approval of the study protocol must also be provided in the manuscript.

Clinical data

Policy information about clinical studies

All manuscripts should comply with the ICMJE guidelines for publication of clinical research and a completed CONSORT checklist must be included with all submissions.

Clinical trial registration	does not apply
Study protocol	does not apply
Data collection	does not apply
Outcomes	does not apply

Flow Cytometry

Plots

Confirm that:

☒ The axis labels state the marker and fluorochrome used (e.g. CD4-FITC).

☒ The axis scales are clearly visible. Include numbers along axes only for bottom left plot of group (a 'group' is an analysis of identical markers).

☒ All plots are contour plots with outliers or pseudocolor plots.

☒ A numerical value for number of cells or percentage (with statistics) is provided.

Methodology

Sample preparation	In brief, 2.7ml of digestion solution containing 400[illegible]l TrypLE Express (Life Technologies), 2000[illegible]l Papain (Worthington; 25U/ml

Sample preparation

in aCSF), 100⍰l DNase I (Worthington; 1mM in aCSF) and 200⍰l Collagenase/Dispase (Roche; 20mg/ml in CS). Vybrant dye (Vybrant Ruby) and NeuO dye (Stem Cell Technology) was added to the digestion mix. Vybrant Dye incorporates into nucleated cells to stain for live neurons while NeuO is a dye used for in vitro enrichment of neurons. We implemented the dye to further enrich for the neuronal cell population.
Ganglia were digested on a heating block at 37°C shaking for 1.5 h. every 30 min the cell suspension was further mechanically disrupted by pipetting up and down starting with a 1 ml pipette going down to a 200 ⍰l pipette. As soon as all ganglion were dissociated the cell suspensions were filtered using a 40mm cell strainer (FALCON) and collected in a 15ml plastic tube. The digestion solution was diluted with 10 ml RPMI medium containing 5% BSA and 1% FCS and centrifuged at 100 g for 4min at 4°C. The supernatant was removed and the pellet resuspended in 200⍰l (CG) and 500⍰l (DRG) RPMI medium containing 5% BSA and 1% FCS.

Instrument

For cell sorting:
FACS Aria I, II and III, FACS Aria Fusion (Becton Dickinson)
For analysis:
LSRII, LSR Fortessa

Software

BD FACSDiva v8.0.3 (Flowcytometers and sorters, BD Bioscience); FlowJo V 10.5.3

Cell population abundance

FB+ Neuron populations abundance was variable depending on the assay. For CG abundance was around 80%, while for DRG it was 0-20% and in JNG around 5-15%.

Gating strategy

For gating of neurons, the gating strategy as depicted in the extended data was:
FSC-A vs. SSC-A --> cells
FSC-A vs. FSC-H --> single cells
FSC-A vs. Vibrant dye --> life cells
FSC-A vs. NeuO --> Neurons
FSC-A vs. FB --> FB + neurons

Enrichment for PDAC- cell subpopulations:
FSC-A vs. SSC-A --> cells
FSC-A vs. FSC-H --> single cells
FSC-A vs. DAPI --> life cells
CD31 vs. CD45 --> CD31+ cell: endothelial, CD45+ cells: Immune cells
EPCAM-Human vs. EPCAM- mouse--> PDAC cells vs. epithelial cells. Double negative cells were sorted as stromal cells

☒ Tick this box to confirm that a figure exemplifying the gating strategy is provided in the Supplementary Information.
